# Extracellular vesicle-mediated delivery of circp53 suppresses the progression of multiple cancers by activating the CypD/TRAP/HSP90 pathway

**DOI:** 10.1038/s12276-025-01506-0

**Published:** 2025-08-01

**Authors:** Xichao Yu, Pinggang Ding, Mengjie Guo, Xiaozhu Tang, Ze Wang, Yuanjiao Zhang, Lianxin Zhou, Xinyu Lv, Hui Shi, Hongming Huang, Jialei Mao, Zhancheng Gu, Chunyan Gu, Ye Yang

**Affiliations:** 1 Kunshan Hospital of Chinese Medicine, Affiliated Hospital of Nanjing University of Chinese Medicine, Kunshan, China; 2 School of Medicine, Nanjing University of Chinese Medicine, Nanjing, China; 3 Department of Hematology, The Affiliated Hospital of Nantong University, Nantong, China

**Keywords:** Diagnostic markers, Translational research, Targeted therapies

## Abstract

The majority of cancers remain incurable due to limited therapeutic responses in malignancies with high-risk genetic mutations such as *TP53*. Building on the success of mRNA vaccine technology, we investigated circular RNA (circRNA) therapeutics and identified hsa_circp53_0041947, a *TP53*-derived circRNA in multiple myeloma (MM). The hsa_circp53_0041947 encodes a functional peptide (circp53–209aa) demonstrating p53 mutation-independent anti-MM effects through CypD/TRAP1/HSP90 complex-mediated mechanisms. Specifically, circp53–209aa activated cyclophilin D (CypD) isomerase activity at the circp53–209aa-R175 site, triggering mitochondrial permeability transition pore opening and subsequent mitochondrial apoptosis. To enable targeted delivery, we engineered extracellular vesicle (EV) systems, E7-Lamp2b-EVs and Her2-Lamp2b-EVs, for MM and colorectal cancer, respectively. Circp53-EVs administration achieved tumor-selective growth inhibition in both malignancies. Our study establishes engineered circp53-EVs as a versatile therapeutic platform, demonstrating the translational potential of circRNA-based strategies for refractory cancers with *TP53* pathway alterations.

## Engineered circRNA-EVs target refractory cancers effectively

Cancer is a major health issue worldwide, and despite advances in treatment, many cancers remain difficult to cure, especially those with high-risk genetic mutations such as *TP53*. Here researchers explore a new approach using circular RNA (circRNA) from the *TP53* gene. They found that a specific circRNA, circp53, could be translated into a functional peptide inducing cancer cell death by interacting with proteins that control cell survival. They developed a method to deliver this circRNA directly to cancer cells using engineered extracellular vesicles, which are tiny particles carrying drugs to specific cells. This targeted delivery system was tested in laboratory experiments and showed promising results in reducing tumor growth in multiple cancer types, including multiple myeloma and colorectal cancer. The study concludes that circp53–209aa has potential as a new treatment for cancers with *TP53* mutations. This summary was initially drafted using artificial intelligence, then revised and fact-checked by the author.

## Introduction

Cancer, as a major global public health challenge, remains the second leading cause of mortality worldwide. Despite substantial progress in early detection, surgical techniques and the development of new drugs such as nucleic acid drugs and chimeric antigen receptor T (CAR-T) cell therapies, the overall outcomes for cancer patients have improved^1,2^. However, many patients with high-risk cancers still have poor overall survival rates, particularly those with high-risk genetic mutations such as *TP53* (ref*.*^3^) and KRAS^4^. For instance, in multiple myeloma (MM), the presence of a del(17p) or *TP53* mutation is considered an indicator of high-risk MM^5^. The most frequently mutated gene, *TP53*, is found in 5–6% of newly diagnosed cases of MM and 45% of patients with relapsed MM, and is the only gene consistently associated with shorter survival rates^6^.

*TP53*, a pivotal tumor suppressor gene within the human genome, encodes the p53 protein located within the minimally deleted region of chromosome 17p13, exhibits mutations in approximately one-third of patients with MM with del(17p)^7^. Notably, del(17p)/*TP53*mut or del(17p)/*TP53*del ‘double-hit’ genetic mutation events are observed in nearly 15% of patients with MM^8^. There is a significant difference in survival rates between patients with and without *TP53* mutations. Those with a *TP53* mutation have a median progression-free survival rate of 5 months and a median overall survival rate of 7 months from detection. In contrast, patients with MM without a detectable *TP53* mutation have a median progression-free survival rate of 11.5 months and a median overall survival rate of 17 months^9^. Beyond hematologic malignancies, *TP53* is the second most frequently mutated gene in colorectal cancer (CRC), occurring in approximately 60% of patients with CRC, and is strongly associated with advanced disease stages and poor prognosis^10^. This mutation also serves as a high-risk biomarker across diverse malignancies, including hepatocellular carcinoma^11^, breast cancer^12^, stomach cancer^13^ and lung cancer^14^. The pervasive clinical impact of *TP53* aberrations underscores the urgent need for targeted therapies, such as small-molecule reactivators of mutant *TP53* or CRISPR-based gene editing strategies, to address this unmet medical challenge.

mRNA vaccines targeting severe acute respiratory syndrome coronavirus 2 (SARS-CoV-2) employ either the full-length spike protein or its receptor-binding domain (RBD) as immunogens, effectively inducing neutralizing antibodies and robust T-cell responses. Developed at an accelerated pace during the pandemic, these vaccines have entered clinical trials while capturing substantial public interest due to their innovative mechanism of action and the urgent global need for effective countermeasures^15,16^. Notably, recent advancements in circular RNA (circRNA) vaccine technology demonstrate superior antigen persistence compared with conventional mRNA vaccines. Researchers have achieved prolonged antigen expression by engineering circRNAs encoding the SARS-CoV-2 RBD, eliciting a potent and durable RBD-specific immune response in vivo^17^. Unlike linear RNAs, which exhibit a relatively short half-life (4.0–7.4 h), circRNAs maintain remarkable stability, with half-lives ranging from 18.8 to 23.7 h, owing to their covalently closed circular structure that resists exonuclease degradation^18^. Their covalently closed structure confers resistance to exonuclease degradation, enabling sustained antigen production^19^. Our research group has previously explored the functional significance of novel circRNA targets in patients with MM. Utilizing Agilent SBC-ceRNA microarray chip sequencing, we identified circCHEK1, circHNRNPU and circBUB1B^16^ as the three most abundant and differentially expressed circRNAs in MM. Intriguingly, our findings demonstrate that these circRNAs possess coding potential, translating into previously uncharacterized peptides that are secreted into the bone marrow microenvironment and which exert regulatory effects on various cell types. Current investigations continue to uncover the therapeutic potential of circRNAs, particularly their advantages in sustained expression and translation capabilities. While existing literature has predominantly focused on circRNA applications in vaccine development^20^ and biosensing technologies^21^, we propose that circRNAs represent a promising and innovative platform for cancer therapy.

Despite the promising potential of circRNAs in vaccine development and biosensing applications, their clinical translation has been notablely limited by the inherent instability of unmodified circRNAs during storage. This challenge is particularly critical, as evidenced by ongoing clinical trials exploring RNA-based therapies; for instance, a phase I study evaluating KRAS G12D siRNA (siKRAS-G12D) delivered via mesenchymal stromal cell-derived extracellular vesicles (EVs) in 28 patients with pancreatic cancer^22^. EVs are gaining increasing attention as highly promising natural drug delivery platforms due to their intrinsic biocompatibility, low immunogenicity and exceptional cargo-carrying capacity^23^. In cancer therapeutics, targeted delivery strategies leveraging EVs aim to enhance drug accumulation in malignant cells while reducing systemic toxicity and off-target effects in healthy tissues^24^. Recent advances in EV engineering have enabled the display of tumor-specific ligands on EV membranes through genetic modification, thereby achieving precision targeting and notablely improving antitumor efficacy^2^. Building upon these developments, the combination of engineered EV delivery systems with the unique structural stability of circRNAs (particularly through appropriate modifications) presents a compelling strategy for advancing circRNA-based cancer therapeutics.

The present study focused on the tumor suppressor *TP53*, a critical regulator frequently inactivated through deletion or mutation in various cancers. Our findings demonstrate that a specific circRNA derived from the *TP53* gene, called circp53 (hsa_circp53_0041947), is substantially downregulated in patients with MM, and its expression correlates with improved clinical outcomes. To explore the therapeutic potential of circp53 in oncology, we developed engineered EVs expressing circp53 and Lamp2b-E7. Our goal is to extend the application of this multifunctional nanoplatform to treat other high-risk and relapsed cancers, including colon cancer, breast cancer, lung cancer and other different types of cancer.

## Materials and methods

### Study design

The overall design of this study was to develop a targeted EV delivery system (E7-Lamp2b-EVs and Her2-Lamp2b-EVs) for delivering circp53 to tumor sites with spatial and temporal selectivity to treat high-risk and refractory cancers. In vitro experiments were conducted to identify the most differentially and abundantly expressed circRNA in healthy controls compared with patients with MM. The methods used included reverse transcription quantitative real-time PCR (RT–qPCR), RNase R digestion, BaseScope RNA in situ hybridization (ISH), plasmid construction, transfection, cell proliferation, colony formation, western blotting (WB), co-immunoprecipitation (Co-IP), immunofluorescence staining, immunohistochemical staining and confocal microscopy, EV isolation and identification, liquid chromatography–mass spectrometry (MS) analysis and cell uptake assays. Targeted EVs were prepared by co-transfection with the plasmids of E7-Lamp2b/Her2-Lamp2b and circp53, followed by differential centrifugation and EV purification. The EVs were characterized by WB, transmission electron microscopy (TEM), and nanoparticle tracking analyses (NTA).

In the MM cell line-derived xenograft (CDX) model, circp53 empty vehicle (Ctrl) and circp53-overexpressing (OE) H929 cells (1 × 10^6^) were injected subcutaneously into the left and right flanks of 8-week-old NOD/SCID mice (*n* = 6) (Gem-Pharmatech LLC.), respectively. Tumor diameter was measured with calipers two to three times weekly. After 28 days, the mice were euthanized by spinal dislocation when the tumor diameter reached 15 mm. The tumors were isolated, weighed and imaged. The tumor volumes were calculated using the formula 0.52 × larger diameter × (smaller diameter)^2^. In the MM patient-derived xenograft (PDX) model, the biopsy samples were collected from an extramedullary tumor subcutaneously under the head skin of a patient with MM at the Department of Hematology, the First Affiliated Hospital of Nanjing Medical University. The NOD/SCID-TIBIA mouse model was established by injecting MM cells into the bone marrow cavity of the tibias of 8-week-old NOD/SCID mice. The tumor accumulation and biodistribution of EVs were evaluated by an in vivo imaging system (IVIS) experiment. The mice were monitored daily and euthanized at defined humane endpoints. All animal experiments were conducted according to a protocol approved by the Institutional Ethics Review Board of Nanjing University of Chinese Medicine (ethics registration no. 201905A003).

### Antibodies and reagents

The primary antibodies used in this study were diluted at a ratio of 1:1,000, including p53 (10442-1-AP, ProteinTech Group), hemagglutinin (HA) (51064-2-AP, ProteinTech Group), cyclophilin D (CypD) (12716-1-AP, ProteinTech Group), Alix (2171S, Cell Signaling Technology), CD9 (13174S, Cell Signaling Technology), TRAP1 (92345, Cell Signaling Technology), HSP90 (13171-1-AP, ProteinTech Group), BCL-xL (2762s, Cell Signaling Technology), Bax (2774s, Cell Signaling Technology), poly-ADP-ribose polymerase (PARP) (9542S, Cell Signaling Technology), cleaved-Caspase 3 (9661S, Cell Signaling Technology) and β-actin (4970S, Cell Signaling Technology). The secondary antibodies included goat anti-Rabbit IgG (H + L), HRP (FMS-Rb01, Fcmacs) or mouse (S0002, Affinity) at a dilution of 1:5,000. The goat anti-rabbit IgG/Alexa Fluor 647 (BS-0295-G, Bioss) and Goat pab to Ms IgG (FITC) (ab6785, Abcam) were used at a dilution of 1:200.

Trizol reagent (10606ES60), HiScript First Strand cDNA Synthesis SuperMix for RT–qPCR (gDNA digester plus) (11121ES60) and SYBR Green PCR Master Mix (11201ES03) were obtained from Yeasen Biotechnology Co. Ltd.

### Cell lines and cell culture

Human MM cell lines, MM1.S, H929, RPMI 8226, XG1, RKO, HCT116, HT29, HepG2, SGC-7901, A549 and MCF-7 cells were cultured in RPMI-1640 (Biological Industries). HEK293 and HCOEPIC cells were cultured in Dulbecco’s modified Eagle medium (Thermo Fisher Scientific). RPMI-1640 and Dulbecco’s modified Eagle medium were supplemented with 10% fetal bovine serum (Gibco), 100 U/ml penicillin and 100 μg/ml streptomycin (HyClone). The cells were cultured at 37 °C in 5% CO_2_.

### BaseScope RNA ISH assay

Circp53 levels in fresh paraffin-embedded tissues from Affiliated Beijing Chaoyang Hospital of Capital Medical University and Affiliated Hospital of Shandong University of Chinese Medicine were analyzed using the BaseScope Reagent kit v2-RED (Advanced Cell Diagnostics), according to the manufacturer’s instructions as previously described.

### Plasmids and transfection

The generation of circRNA was conducted according to the previous protocol^23^. The circRNA database (http://reprod.njmu.edu.cn/cgi-bin/circrnadb/circRNADb.php) was adopted to predict the open reading frame (ORF) of circp53. A commercially available circRNA expression vector, PLC5-ciR (GS0104, Guangzhou Geneseed Biotech Co.), was used to construct a circp53-overexpression vector based on the predicted translation mode. To induce circularization in vivo, side-flanking repeat sequences and SA/SD sequences were added to both sides of the 858 nt sequences with oncolytic virus (OV-circp53). The front circular frame contained the endogenous flanking genomic sequences with an EcoRI restriction enzyme site, while the back circular frame included part of the inverted upstream sequence with a BamHI site. Cancer cells were transfected using lentivirus, as previously described.

### Cell proliferation, colony formation and cell apoptosis assays

Cell proliferation, colony formation and cell apoptosis were examined using MTT, soft agar colony formation and annexin V/PI staining, as described previously.

### EdU incorporation assay

The 5-ethynyl-2′-deoxyuridine (EdU) Cell Proliferation kit (C0071S, Beyotime) was utilized to label proliferating cells. The cells were fixed with 4% paraformaldehyde at 4 °C for 25 min. Click additive was used to prepare the click reaction solution, which was then incubated for 30 min at room temperature in the dark. The sample was then rinsed with PBS two to three times. Next, a 2 μg/ml 4,6-diamidino-2-phenylindole (DAPI) solution was incubated for 5 min at room temperature, followed by moistening the samples with PBS two to three times.

### TUNEL assay

The terminal deoxynucleotidyl transferase-mediated dUTP-biotin nick end labeling (TUNEL) Apoptosis Detection kit (YSFluorTM 640, Yeasen) was used to label apoptotic cells. The cells were fixed with 4% paraformaldehyde at 4 °C for 25 min. To prepare the Proteinase K solution, a 1:100 ratio of 2 mg/ml Proteinase K to PBS was used to achieve a final concentration of 20 μg/ml. A volume of 100 μl of this solution was added to each sample, followed by incubation at room temperature for 3 min. The samples were then rinsed with PBS two to three times. Next, 100 μl of 1× equilibration buffer was added to each sample to completely cover the sample area to be detected and the samples were incubated at room temperature for 30 min. After most of the 1× equilibration buffer was washed off with absorbent paper, 50 μl of TdT incubation buffer was added. The slide was placed in a wet box and incubated at 37 °C for 60 min. The sample was then rinsed with PBS two to three times and incubated with 2 μg/ml DAPI solution for 5 min at room temperature. Finally, the sample was rinsed with PBS two to three times.

### Transfection and preparation of EVs

HEK293 cells were cotransfected with the circp53 lentivirus and E7-Lamp2b lentivirus to generate E7-Lamp2b-circp53-OE cells. Similarly, HEK293 cells were cotransfected with the circp53 lentivirus and Her2-Lamp2b lentivirus to create Her2-Lamp2b-circp53-OE cells. After 48 h, puromycin was added to the cell culture medium to select successfully transfected cells. E7-circp53-EVs and Her2-circp53-EVs were prepared from the supernatant fluids of HEK293 E7-Lamp2b-circp53-OE cells and HEK293 Her2-Lamp2b-circp53-OE cells, respectively. Circp53-EVs were isolated from the supernatant fluids of HEK293 circp53-OE cells used as controls. The medium was centrifuged at 300*g* for 5 min to remove cells. The next centrifugation step was performed at 10,000*g* for 60 min to remove shedding vesicles, followed by centrifugation at 3000*g* for 30 min to remove cell debris. The supernatant was filtered with a 0.22 μm pore filter and centrifuged at 200,000*g* for 90 min. The pellets were resuspended with PBS following the centrifugation step at 200,000*g* to purify EVs.

### Characterization of EVs

We used the WB method to detect the presence of Alix and CD9 proteins in purified EVs. The morphology of EVs was characterized using a JEM-2100 transmission electron microscope (JEOL). The size distribution of the particles was measured by NTA.

### EVs staining and dynamic cellular uptake imaging

The PKH26 Linker kit (MX4021, MKbio) was used to label EVs to observe the cellular uptake of targeted EVs, according to the manufacturer’s instructions. The EVs were washed with a serum-free medium and resuspended in 1 ml of Diluent C. To create a 2× staining solution, 4 μl of PKH26 ethanol storage solution were added to 1 ml of Diluent C and thoroughly mixed. The EVs were immediately mixed with a pipette in this solution, resulting in a final PKH26 concentration of 2 μM. The EV suspension was incubated at 25 °C for 5 min with regular mixing. Next, 2 ml of fetal bovine serum was added and the mixture was incubated at 25 °C for an additional 5 min to combine with any excess probes. The suspension was filtered through a 0.22 μm pore filter and centrifuged at 200,000*g* for 90 min to obtain PKH26-labeled EVs. These labeled EVs, specifically E7-circp53-EVs, Her2-circp53-EVs or circp53-EVs, were resuspended in a serum-free medium at 200 μg and added to the corresponding cells at 37 °C. After incubation, the cells were washed with PBS two to three times to remove any excess EVs and prepared for subsequent experiments.

### Immunofluorescence staining and confocal imaging

Immunofluorescence staining was conducted using the method described previously. A confocal microscope (TCS SP8, Leica) was utilized to capture images.

### NOD/SCID-TIBIA mouse model

MM1.S cells were injected into the bone marrow cavity of the tibias of 8-week-old NOD/SCID mice as described previously.

### Intravenous delivery of circp53 in vivo

E7-circp53-EVs and Her2-circp53-EVs were administered intravenously at a dose of 5 mg/kg. To assess the in vivo biodistribution of overexpressed circp53, we used IVIS to monitor the targeted delivery efficiency.

### Statistical analyses

Statistical analyses were performed using SPSS version 22.0 or GraphPad Prism 8.01 software, and all values were expressed as mean ± s.d. unless otherwise specified. A two-tailed Student’s *t*-test (two groups) or one-way analysis of variance with Tukey’s post hoc comparison (≥3 groups) was used to assess statistical significance. A Kaplan–Meier curve and Log-rank test were utilized to determine MM patient survival. *P* < 0.05 was considered statistically significant.

## Results

### Circp53 expression is decreased in patients with MM and is associated with superior outcomes

**Fig. 1:**
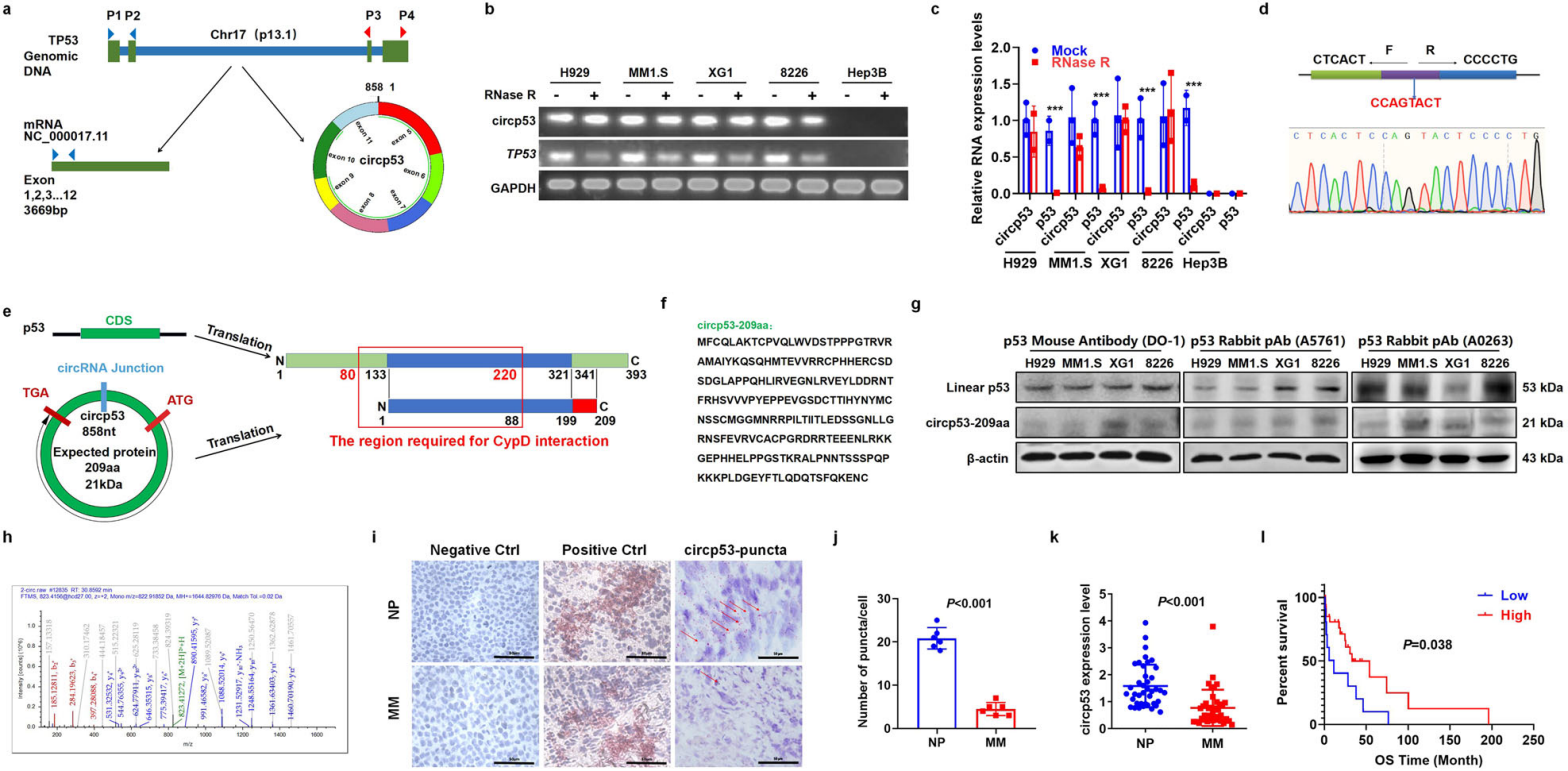
Circp53 expression is decreased in patients with MM and is associated with superior outcomes. **a**, An illustration of the annotated genomic region of *TP53*, the putative different RNA splicing forms and the validation strategy for circular exons 5 and 11 (circp53). Convergent (blue) and divergent (red) primers were designed to amplify the linear or back-splicing products. **b**, RNA levels of circp53 and linear *TP*53 were determined by PCR with and without RNase R treatment. **c**, RNA levels of circp53 and linear *TP53* were determined by RT–qPCR with and without RNase R treatment. **d**, Sanger sequencing following PCR was conducted using the indicated divergent flanking primers, confirming the ‘head-to-tail’ splicing of circp53 in MM1.S, H929, XG1 and RPMI 8226 cells. **e**, The putative ORF in circp53. The sequences of the putative ORF are shown in green. **f**, The predicted sequence of circp53–209aa. **g**, WB analysis of endogenous circp53–209aa expression in MM1.S, H929, XG1 and RPMI 8226 cells. **h**, The specific peptides from circp53–209aa were identified by MS analysis. **i**, The levels of circp53 in patients with MM were lower than in those in NP as evaluated by BaseScope analysis and the representative staining images are shown with positive reactions indicated by red arrows. **j**, Statistical analysis of BaseScope analysis (n = 6 clinical samples for each group, *P* < 0.001). **k**, Circp53 levels were significantly decreased in patients with MM (*n* = 48 samples for each group, *P* < 0.001). **l**, Higher levels of circp53 were associated with longer overall survival (OS) survival. The data are presented as mean ± s.d. ^*^P < 0.05, ^**^P < 0.01 and ^***^P < 0.001.

*TP53*, a critical tumor suppressor gene located on chromosome 17p13.1, has been extensively studied for its role in maintaining genomic integrity. To investigate the potential formation of a circRNA from the *TP53* gene, we designed specific primers targeting both linear and back-spliced regions of the p53 transcript (hsa_circ_0041947). A back-spliced product confirmed the existence of a circRNA derived from exons 5 and 11 of the *TP53* gene (Fig. 1a).

To confirm the presence of circp53, we conducted RNase R digestion assays, which selectively degrade linear RNAs while leaving circRNAs intact. The results showed significant degradation of linear *TP53* transcripts (H929, *P* < 0.001; MM1.S, *P* < 0.05; XG1, *P* < 0.01, RPMI 8226: *P* < 0.01). In contrast, circp53 exhibited remarkable resistance to RNase R treatment, confirming its stable existence in both wild-type (WT) p53 (H929 and MM1.S) and mutant p53 (XG1 and RPMI 8226) cell lines (Fig. 1b,c). As negative controls, we employed Hep3B cells (p53-null), with their p53-deficient status being verified by WB analysis and EdU incorporation assays (Supplementary Fig. 1). Furthermore, Sanger sequencing was performed to precisely identify the circp53 back-splice junction site (Fig. 1d).

Numerous studies have demonstrated that circRNAs have the potential to be translated into proteins or peptides that play essential roles in biological functions. Our previous studies have shown that circCHEK1 (ref.^16^), circHNRNPU^25^ and circBUB1B^26^ are translatable, and they can be secreted into the bone marrow niche, making them potential diagnostic markers and potential therapeutic targets for MM. Initially, we analyzed the ORF of circp53 by using the circRNADb database. Interestingly, we discovered that an internal ribosome entry site sequence (from +419 to +567 nt) of circp53 within the ORF that encodes a 209-amino-acid (aa) peptide, which we have named ‘circp53–209aa’. This novel peptide contains 87 aa (aa 133–220) that correspond to the linear p53 sequence, which is known to interact with the mitochondrial permeability transition pore (mPTP) regulator CypD^11^ (Fig. 1e, f). To confirm the potential of endogenous circp53 to encode circp53–209aa, we used a commercial p53 antibody to detect the N-terminus of p53. WB analysis successfully confirmed the presence of circp53–209aa at the expected molecular size (21 kDa) in MM1.S, H929, XG1 and RPMI 8226 cells (Fig. 1g). Liquid chromatography with a tandem MS system was also used to identify specific peptide fragments from circp53–209aa, further confirming the existence of circp53–209aa (Fig. 1h).

To further support our findings, we conducted a study using clinical samples. We used a BaseScope RNA ISH assay with a commercial circRNA junction probe to detect the expression levels of circp53 in tissues from patients with MM. We also used the human housekeeping gene *PPIB* as a positive control probe and the bacterial gene *dapB* as a negative control probe. Our results showed a significant decrease in circp53 levels in MM tissues (Fig. 1I, j) compared with those in healthy controls (normal person, NP) (*P* < 0.001). Furthermore, we collected blood samples from 48 patients with MM and 48 NP to assess circp53 expression levels. We found a marked decrease in circp53 levels in MM samples compared with NP (*P* < 0.001), and patients with MM with higher circp53 expression had longer overall survival rates (Fig. 1k, l). These findings suggest that decreased circp53 levels may be associated with better outcomes in patients with MM.

### Circp53–209aa activates the mitochondrial apoptotic pathway

**Fig. 2:**
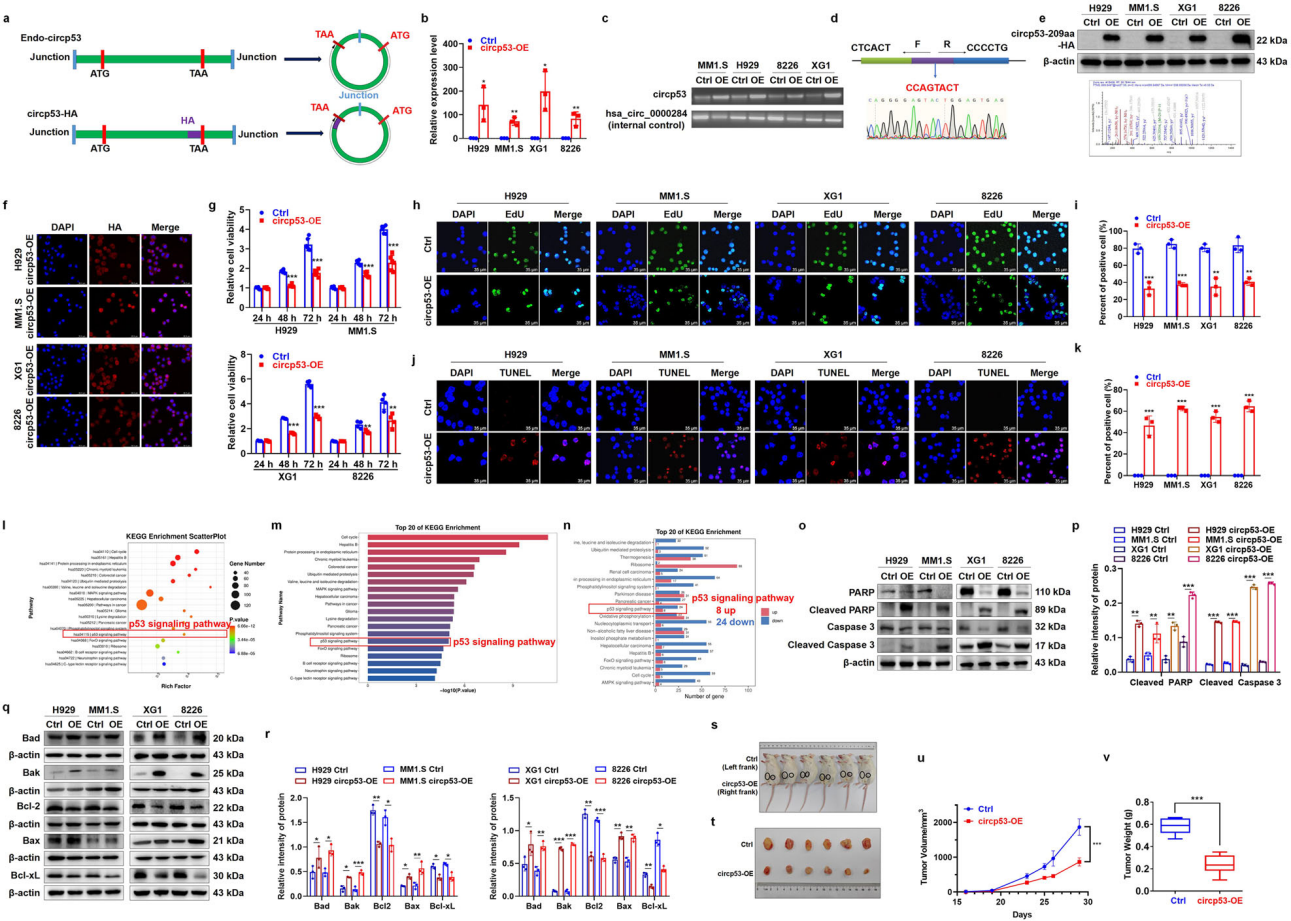
Circp53 activates the mitochondrial apoptotic pathway. **a**, An illustration of endo-circp53 and circp53-HA. **b**, RT-qPCR was used to determine the RNA levels of circp53 and linear *TP*53. **c**, PCR was used to determine the RNA levels of circp53 and *TP*53. **d**, Sanger sequencing was performed following PCR using the indicated divergent primers to confirm the precise splicing of the circp53-OE plasmid in MM1.S, H929, XG1, and RPMI 8226 cells. The circRNA hsa_circ_0000284 was used as an internal control gene. **e**, WB and MS analyses of circp53–209aa overexpression in MM1.S, H929, XG1 and RPMI 8226 cells using the HA-tag antibody. **f**, Confocal microscope live images showing the cellular localization of circp53–209aa. **g**, The MTT assay demonstrated decreased cell proliferation in circp53-OE cells compared with Ctrl cells (*P* < 0.01). **h**, The EdU incorporation assay revealed a significant decrease in the number of proliferating cells in circp53–209aa-OE cells compared with Ctrl cells. **i**, Statistical analysis of EdU incorporation assay. **j**, The TUNEL assay showed a significant increase in the number of apoptotic cells in circp53–209aa-OE cells compared with Ctrl cells. **k**, Statistical analysis of EdU incorporation assay. **l**, The scatterplot of KEGG pathway enrichment analysis for RNA-sequencing data revealed that the molecular function of circp53–209aa was centered on the p53 signaling pathway. **m**, The par chart of KEGG pathway enrichment analysis for RNA-sequencing data also revealed that the molecular function of circp53-209aa was centered on the p53 signaling pathway. **n**, The KEGG enrichment analysis revealed that the p53 signaling pathway was enriched, ranking within the top 20 pathways. **o**, WB analysis demonstrated that increased levels of circp53 induced apoptosis, as indicated by the cleavage of the apoptotic regulators PARP and Caspase 3. **p**, Statistical analysis of the expression of cleavage of the apoptotic regulators PARP and Caspase 3. **q**, WB analysis determined the expression levels of the mitochondrial apoptotic pathway associated proteins Bad, Bak, Bcl-xL, Bax and Bcl-2 in circp53-OE cells. **r**, Statistical analysis of the expression of the mitochondrial apoptotic pathway associated proteins. **s**, Images of CDX model mice on day 28. **t**, Tumors taken from the CDX model in each group. **u**, The tumor growth curve of the model mice in the Ctrl and circp53-OE groups. **v**, The tumor weight of the CDX model mice in the Ctrl and circp53-OE groups. The data are presented as mean ± s.d. (*n* = 6 mice for each group, *n* = 3 cultures for each group, ^*^P < 0.05, ^**^P < 0.01 and ^***^P < 0.001).

To investigate the molecular function of circp53–209aa, the full-length sequence of circp53–209aa was inserted into the commercially available circRNA expression vector PLC5-ciR (GS0104; Guangzhou Geneseed Biotech Co. Ltd.) and linked with an HA tag (Fig. 2a). The plasmid was transfected into MM1.S, H929, XG1 and RPMI 8226 cells using a lentivirus system. RT–qPCR assay confirmed that circp53–209aa was indeed overexpressed in the four cell lines (Fig. 2b, c). Sanger sequencing was performed to verify the accuracy of the cyclization product (Fig. 2d). WB and MS analyses were then employed to further validate that the specific peptide fragments originated from circp53–209aa (Fig. 2e). In addition, immunofluorescence experiments were performed to identify the cellular localization of circp53–209aa by using an HA antibody, which revealed that circp53–209aa was predominantly found in the cytoplasm (Fig. 2f).

MTT and EdU incorporation assays were performed to determine whether circp53–209aa could function as a tumor suppressor in circp53–209aa-OE cells. Intriguingly, the cell proliferation rate was significantly decreased in circp53–209aa-OE cells compared with the control (Ctrl) wild-type cells (Fig. 2g–i). TUNEL assay also showed a significant increase in apoptotic cells in circp53–209aa-OE cells compared with Ctrl cells (Fig. 2j, k). To investigate the mechanism by which circp53–209aa promotes MM malignancy, we conducted RNA-sequencing on MM cells. The Kyoto Encyclopedia of Genes and Genomes (KEGG) pathway enrichment analysis revealed significant activation of the p53 signaling pathway upon circp53 overexpression (Fig. 2l, m and Supplementary Fig. 2a–c). Importantly, this finding was further validated in additional tumor cell lines, including HepG2 and HCT116 cells, where circp53 overexpression similarly upregulated the p53 signaling pathway (Supplementary Fig. 2d,e), suggesting a conserved tumor-suppressive mechanism across different cancer types. Further analysis showed 8 upregulated and 24 downregulated genes in MM1.S circp53-OE cells compared with MM1.S WT cells (Fig. 2n and Supplementary Table 1), as well as 6 upregulated and 10 downregulated genes in H929 circp53-OE cells compared with H929 WT cells (Supplementary Table 2). Similarly, 5 upregulated and 24 downregulated genes were found in RPMI 8226 circp53-OE cells compared with RPMI 8226 WT cells (Supplementary Table 3), and 5 upregulated and 26 downregulated genes were found in XG1 circp53-OE cells compared with XG1 WT cells (Supplementary Table 4). These results suggest that the molecular function of circp53–209aa is centered on the p53 signaling pathway. As p53 can induce the mitochondrial apoptosis pathway through mitochondrial membrane permeabilization by interacting with and antagonizing the anti-apoptotic proteins Bcl-xL and Bcl-2 (ref.^27^), we continued to explore whether circp53–209aa could also induce mitochondrial apoptosis. Our experiments showed that the levels of cleaved-PARP and cleaved-caspase 3 were increased in circp53–209aa-OE MM cells compared with Ctrl cells (Fig. 2o, p). Additionally, the levels of Bcl-2 and Bcl-xL were markedly decreased, while the levels of the effector proteins Bak, Bad and Bax were significantly increased in circp53–209aa-OE MM cells compared with Ctrl cells (Fig. 2q, r).

To further investigate the role of circp53 in vivo, we utilized a myeloma CDX model. First, 1 × 10^6^ Ctrl and circp53-OE H929 cells were injected subcutaneously into six NOD/SCID mice, a more immunosuppressive mouse model, to establish another cell-derived xenograft model. Once the tumor diameter reached 15 mm, the mice were killed. Cells were injected subcutaneously into the abdomen of mice: the Ctrl group (left flank) and the circp53-OE group (right flank), and the tumor volume was observed after 4 weeks (Fig. 2s, t). After successfully establishing the model, we observed that the growth rate of tumors in the circp53-OE group was significantly slower than in the Ctrl group over 4 weeks (Fig. 2u). In addition, the average volume (*P* < 0.001) and weight (*P* < 0.001) of the tumors in the circp53-OE group were significantly lower than those in the EV group after dissection (Fig. 2v).

### Combining circP53–209aa with BTZ synergistically inhibits the proliferation of MM cells by activating the mitochondrial apoptotic pathway

**Fig. 3:**
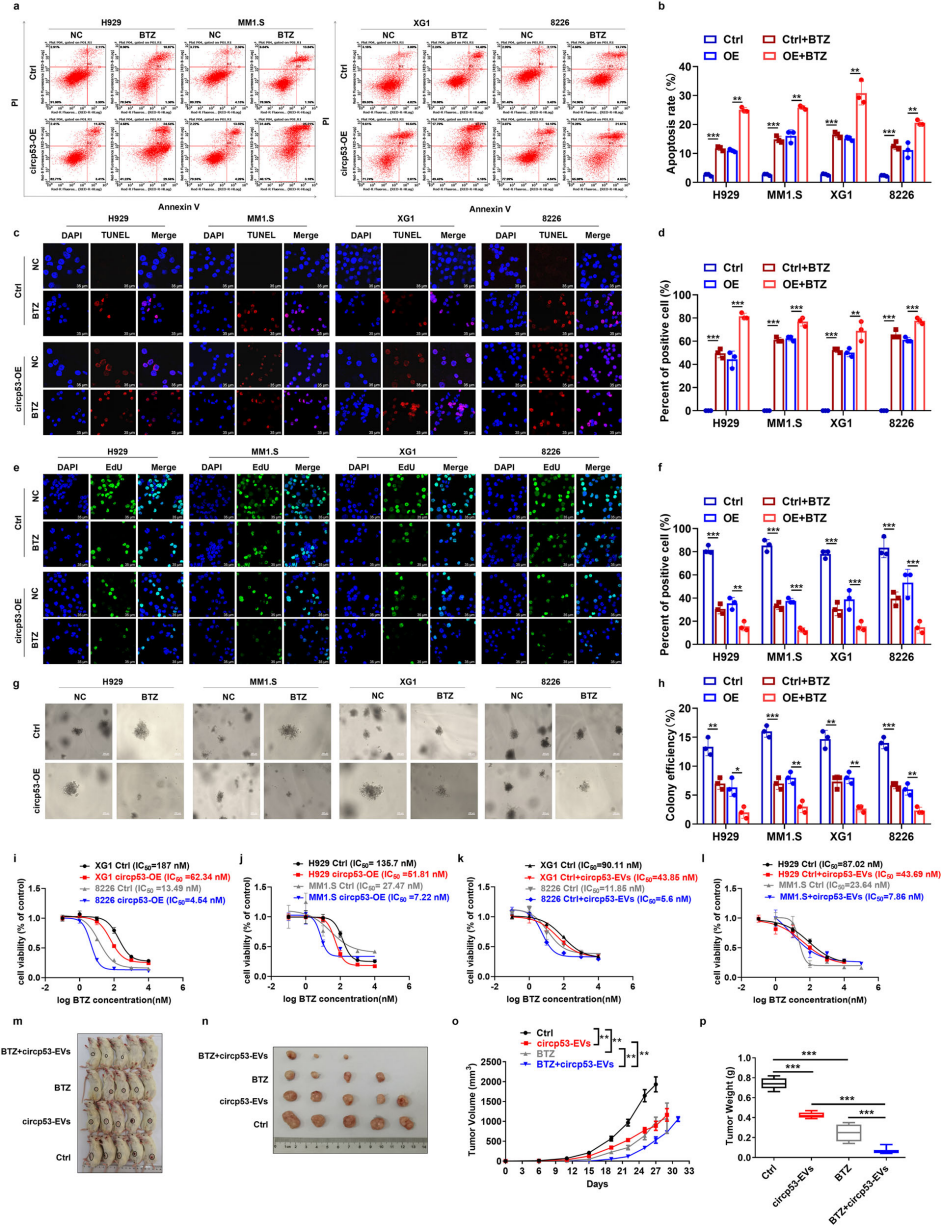
Combining circP53–209aa with BTZ synergistically inhibits the proliferation of MM cells by activating the mitochondrial apoptotic pathway. **a**, The effects of BTZ on cell apoptosis in MM cells with or without circp53 overexpression. The circp53-OE cells were more sensitive to BTZ treatment compared with WT cells (*P* < 0.01). **b**, Statistical analysis of cell apoptosis. **c**, Representative images from the TUNEL assay demonstrate a significant increase in the number of apoptotic cells in the circp53-OE group compared with the Ctrl group after BTZ treatment (*n* = 3 cultures for each group, *P* < 0.01). **d**, Statistical analysis of TUNEL assay. **e**, Representative images from the EdU incorporation assay show a significant decrease in the number of proliferating cells in the circp53-OE group compared with the Ctrl group upon BTZ treatment (*n* = 3 cultures for each group, *P* < 0.01). **f**, Statistical analysis of EdU incorporation assay. **g**, Representative images from the soft agar plates demonstrate a significant decrease in colony-forming efficiency in the circp53-OE group compared with the Ctrl group upon BTZ treatment. **h**, Statistical analysis of colony-forming efficiency (*n* = 3 cultures for each group, *P* < 0.01). **i**, The MTT assay results showed that elevated levels of circp53–209aa significantly increased the sensitivity of p53 mutant cells (XG1 and RPMI 8226) to BTZ treatment. **j**, The MTT assay results showed that elevated levels of circp53–209aa significantly increased the sensitivity of p53 WT cells (MM1.S and H929) to BTZ treatment. **k**, Treatment with EVs overcame BTZ resistance in p53 mutant cells such as XG1 and RPMI 8226 cells. **l**, Treatment with EVs overcame BTZ resistance in p53 WT cells such as H929 and MM1.S cells. **m**, Photographic images of PDX model mice. **n**, Photographic images of PDX model tumors. **o**, The combination of circp53-EVs and BTZ significantly inhibited mean tumor volume. **p**, The combination of circp53-EVs and BTZ significantly inhibited mean tumor weight (*P* < 0.001). The data are presented as mean ± s.d. (*n* = 5 mice for each group, *n* = 3 cultures for each group, *P < 0.05, **P < 0.01 and ***P < 0.001). NC, normal control.

The flow cytometry results showed that circp53–209aa-OE cells had a higher rate of apoptosis compared with Ctrl cells when treated with bortezomib (BTZ) (Fig. 3a, b). The TUNEL assay also revealed that circp53–209aa-OE cells had more apoptotic cells compared with the Ctrl group when treated with BTZ (Fig. 3c, d) and the EdU incorporation assay demonstrated that circp53–209aa-OE cells had fewer proliferating cells than Ctrl cells when treated with BTZ (Fig. 3e, f). To assess the long-term proliferative capacity of MM cells, a soft agar colony formation assay was performed, which showed that circp53-OE MM cells formed fewer colonies than Ctrl cells when treated with BTZ (Fig. 3g, h). Furthermore, the increased level of circp53–209aa enhanced the sensitivity of both p53 WT cells (MM1.S and H929 cells) and p53 mutant cell lines (XG1 and RPMI 8226 cells) to BTZ (Fig. 3I, j). The *N*^6^-methyladenosine (m6A) methylation level exhibits a negative correlation with the translation efficiency of noncoding RNAs. Using the online prediction tool SRAMP, we identified two putative m6A methylation sites in circp53 at positions 486 and 501 (Supplementary Fig. 3a). Comparative analysis revealed that HEK293 cells display significantly lower m6A methylation levels than tumor cells (Supplementary Fig. 3b), which correlates with reduced circp53–209aa expression in HEK293 cells (Supplementary Fig. 3c). Functional assessments using EdU incorporation and TUNEL assays demonstrated that circp53 overexpression did not significantly alter the proliferation or apoptosis rates of HEK293 cells compared with the Ctrl group (Supplementary Fig. 3d,e). We then extracted EVs from circp53-OE HEK293 cells and observed their effect on the proliferation of MM cells. Furthermore, a combination treatment of EVs and BTZ significantly inhibited the cellular proliferation of MM cells, regardless of the presence or absence of p53 mutation (Fig. 3k, l).

To further evaluate the inhibitory effect of circp53-EVs, we employed a myeloma PDX model, which represents one of the most advanced in vivo preclinical tumor models owing to its ability to accurately reproduce patient tumor behavior and therapeutic responses^28^. In this model, patient-derived primary human MM xenografts were implanted into NOD/SCID mice, as previously described. Initial assessment using the CCK-8 assay revealed that circp53-EVs exerted a concentration-dependent suppression of cancer cell proliferation (Supplementary Fig. 4a). Based on the calculated half-maximum inhibitory concentration (IC_50_) values, we performed a dose equivalency conversion to determine the appropriate intravenous injection dosage for subsequent in vivo experiments. The treatment of circp53-EVs at a dose of 5 mg/kg impeded MM cell proliferation in vivo compared with the Ctrl group (Fig. 3m, n). Moreover, the combination treatment of circp53-EVs (5 mg/kg, every other day) and BTZ (1 mg/kg, twice per week) resulted in smaller tumors compared with the single treatment group (Fig. 3m, n). The average volume (*P* < 0.01) and weight (*P* < 0.001) of the tumors in the combination treatment group were also significantly decreased compared with the single treatment group (Fig. 3o, p). Analysis of the malignant proliferation marker Ki67 and the cell proliferation-related protein c-Myc revealed that their expression levels were significantly reduced in the treatment group compared with the Ctrl group (Supplementary Fig. 4b,c). To assess the potential toxicity of circp53-EVs, we administered them via tail vein injection either weekly at a dose of 5 mg/kg for four consecutive weeks or as a single high dose of 50 mg/kg. Comprehensive histological analysis, including hematoxylin and eosin staining of major organs (heart, liver, spleen and kidney), along with immunohistochemical assessment of the inflammatory marker TNF (Supplementary Fig. 4d,e), demonstrated no detectable toxicity in any of the examined tissues. These findings suggest that circp53–209aa can effectively activate the mitochondrial apoptosis pathway and exhibit synergistic antitumor effects when combined with BTZ treatment.

### Circp53–209aa-R175 is identified as the site-specific target of CypD to trigger mPTP opening in MM cells

**Fig. 4:**
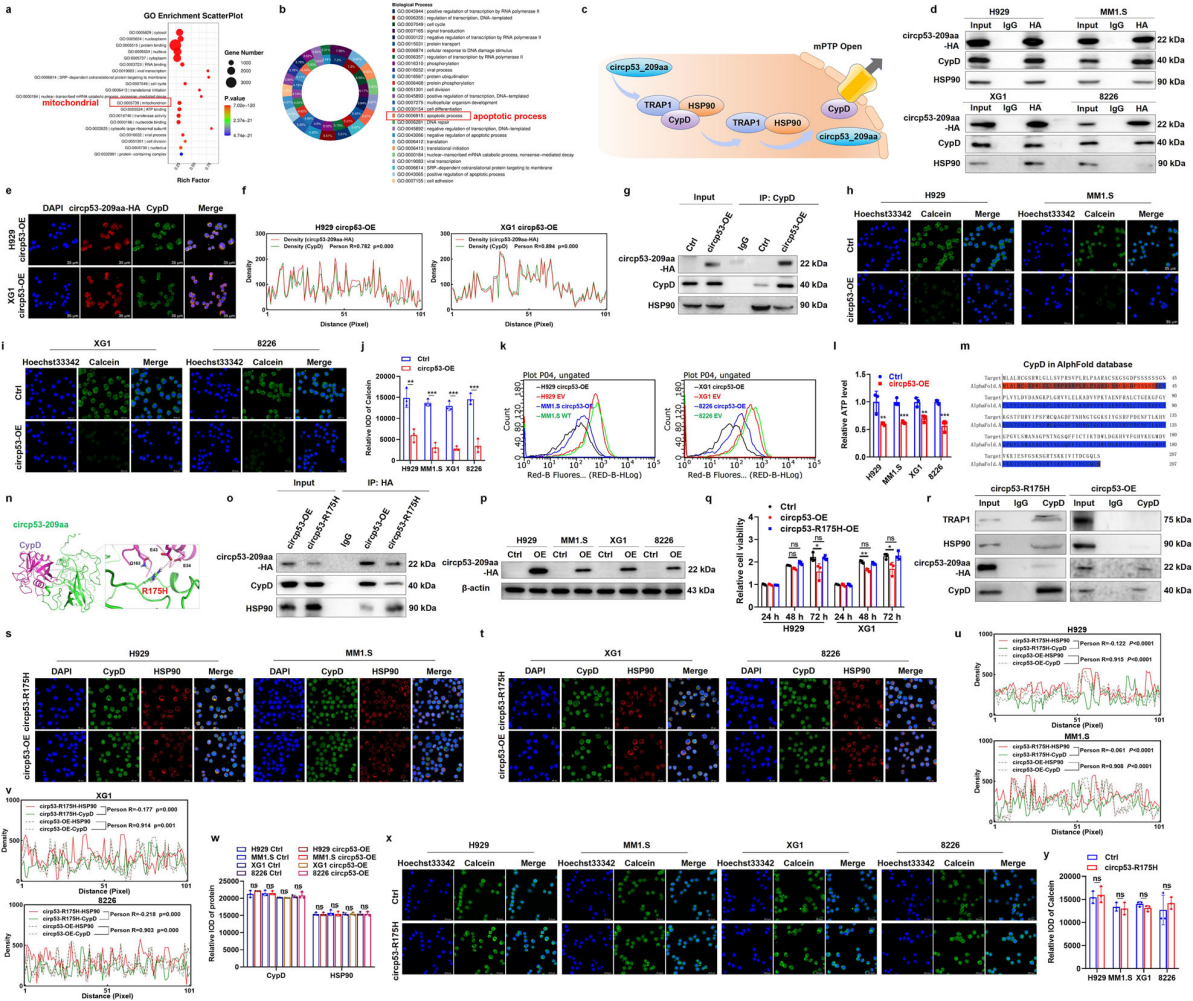
Circp53–209aa-R175 is identified as the site-specific target of CypD to trigger mPTP opening in MM cells. **a**, GO analysis of RNA-sequencing data revealed that the circp53–209aa is highly associated with mitochondrial pathways. **b**, Go analysis of RNA-sequencing data revealed that the circp53–209aa is highly associated with apoptotic process signaling pathways. **c**, A graphic illustration of circp53–209aa interacting with CypD and releasing CypD from the CypD/TRAP1/HSP90 complex, thereby activating its isomerase activity and inducing mPTP opening. **d**, A Co-IP assay confirmed the direct interaction between circp53–209aa and CypD in MM cells. **e**,Representative confocal images of HA and CypD demonstrate the interaction between circp53–209aa and CypD. **f**, Statistical analysis of the colocation of circp53-209aa and CypD in H929 and XG1 cells. **g**, A Co-IP assay showed a weaker interaction between CypD and HSP90 in circp53-OE MM cells compared with Ctrl cells. **h**, Distribution of calcein in H929 and MM1.S cells. **i**, Distribution of calcein in XG1 and RPMI 8226 cells. **j**, IOD quantitative statistics of Calcein in Ctrl and circp53–209aa-OE MM cells. **k**, ATP levels were significantly decreased in circp53–209aa-OE cells. **l**, Statistical analysis of ATP levels in different cells (*P* < 0.001). **m**, A model showing the high confidence interval of the CypD protein. **n**, A model of the circp53–209aa-CypD complex. R175 of circp53–209aa binds to E43, E34 and Q163 of CypD through hydrogen bonds and/or ionic interactions. **o**, Circp53–209aa-R175 is involved in hydrogen-bond interactions with CypD, and these interactions are disrupted upon mutation of arginine to a histidine, as demonstrated by Co-IP using an HA antibody as bait in circp53-OE cells, followed by WB analysis. **p**, Circp53–209aa-R175H-OE MM cells were constructed successfully, as shown by WB analysis. **q**, Circp53–209aa-R175H did not influence the proliferation of MM cells. **r**, Circp53–209aa interacts with CypD and releases it from the CypD/TRAP1/HSP90 complex, as evidenced by Co-IP. **s**, Colocalization dynamics of CypD/HSP90 in H929 and MM1.S cells. **t**, Colocalization dynamics of CypD/HSP90 in XG1 and 8226 cells. **u**, Statistical analysis of the colocation of CypD and HSP90 in H929 and MM1.S cells. **v**, Statistical analysis of the colocation of CypD and HSP90 in XG1 and RPMI 8226 cells. **w**, IOD quantitative statistics of CypD and HSP90 in MM cells. **x**, Distribution of calcein in circp53-OE and circp53–209aa-R175 MM cells. **y**, The mPTP was not opened in the circp53–209aa-R175H group by quantitative statistics. The data are presented as mean ± s.d. (*n* = 3 cultures for each group, *P < 0.05, **P < 0.01, ***P < 0.001 and ns, no statistical significance).

To further investigate the regulatory mechanism of circp53–209aa, we conducted a Gene Ontology (GO) analysis. The results showed a strong association between circp53–209aa and the mitochondrial and apoptotic signaling pathways (Fig. 4a, b), which is consistent with the data presented in Fig. 2. Previous studies have shown that the region of p53 that interacts with the permeability transition pore (PTP) regulator CypD is located within the region of aa 80–220. This interaction triggers the opening of mPTP. As circp53–209aa contains the same region, we hypothesized that it may also interact with CypD, releasing it from the CypD/TRAP1/HSP90 complex and activating its isomerase activity^29^, ultimately leading to mPTP opening (Fig. 4c). We also performed a Co-IP assay using an HA antibody as bait to confirm the interaction between circp53–209aa and CypD in MM cells (Fig. 4d). In addition, immunolocalization analysis showed that circp53–209aa interacts and colocalizes with CypD (Fig. 4e, f). To determine whether circp53-OE MM cells are more likely to induce apoptosis through the CypD/TRAP/HSP90 pathway, we conducted a Co-IP assay using a CypD antibody as bait, which confirmed that the interaction between CypD and HSP90 is weaker in circp53-OE MM cells compared with Ctrl cells (Fig. 4g). The fluorescent dye calcein, a highly selective indicator of sustained mPTP opening, was utilized for immunofluorescence experiments to further examine the effect of circp53–209aa-induced alterations in the function of mPTP^30^. As expected, the confocal microscopic and flow cytometry analyses showed a rapid and almost complete loss of fluorescence signal in circp53–209aa-OE cells compared with Ctrl cells (Fig. 4h–j). Furthermore, we observed that an increased level of circp53–209aa led to decreased ATP levels in MM cells (Fig. 4k, l). These results suggest that circp53–209aa may trigger mPTP opening through physical interaction with the PTP regulator CypD.

A protein–protein docking experiment investigated the interaction between circp53–209aa and CypD. The AlphaFold-predicted model revealed a high-confidence structural representation of CypD (blue) (Fig. 4m). Notably, R175 of circp53–209aa was found to form hydrogen bonds and/or ionic interactions with E43, E34 and Q163 of CypD (Fig. 4n). The *TP53* (R175) mutation is a well-known ‘hotspot’ for p53 mutations in various types of cancer^31^. To further validate circp53–209aa-R175 as a site-specific binding target of CypD, a Co-IP assay was performed, which revealed a strong interaction between CypD and circp53–209aa. Strikingly, this interaction was markedly diminished in HEK293 cells expressing circp53–209aa-R175H (Fig. 4o). Consistent with these observations, Reichert 4SPR analysis demonstrated that the R175H mutation significantly impaired the binding affinity between circp53–209aa and CypD (Supplementary Fig. 5). To further explore the functional implications of this interaction, we generated MM cells stably overexpressing circp53–209aa-R175H via lentiviral transduction, as confirmed by WB analysis (Fig. 4p). The result of the MTT assay showed no significant difference in cellular proliferation between Ctrl and circp53–209aa-R175H-OE cells (Fig. 4q). However, it was found that circp53–209aa, rather than circp53–209aa-R175H, interacts with CypD, leading to the release of CypD from the CypD/TRAP1/HSP90 complex, thereby activating its isomerase activity and inducing mPTP opening, as evidenced by the Co-IP assay (Fig. 4r). Although there were significant differences in the localization of fluorescence signals comparing circp53–209aa-R175H-OE and circp53–209aa-OE cells (Fig. 4s–v), there was no significant difference in the expression of fluorescence signals (Fig. 4w). Furthermore, immunofluorescence staining showed no significant difference in the expression of Calcein, indicating that the mPTP was not opened in Ctrl and circp53–209aa-R175H-OE cells (Fig. 4x, y). These experiments suggest that circp53–209aa-R175 can competitively interact with CypD from the CypD/TRAP1/HSP90 complex, thereby activating its isomerase activity and inducing mPTP opening. Therefore, circp53–209aa may be a potential alternative approach for restoring p53 activity.

### CircP53–209aa activates the mitochondrial apoptosis pathway in colorectal, lung, stomach, liver and breast cancer cells

**Fig. 5:**
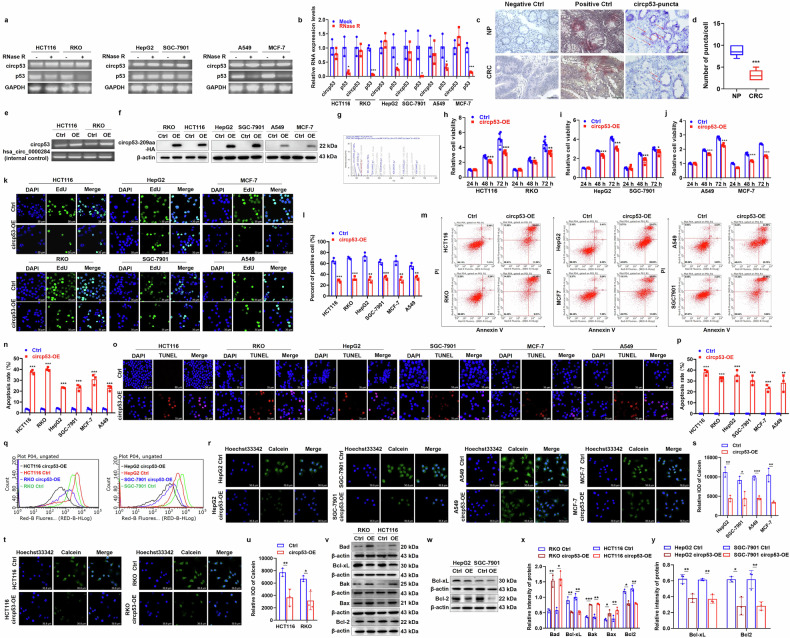
Circp53–209aa activates the mitochondrial apoptosis pathway in colorectal, lung, stomach, liver and breast cancer cells. **a**, The RNA levels of circp53 and linear p53 were determined by RT–PCR with and without RNase R treatment in RKO, HCT116, HepG2, SGC-7901, A549 and MCF-7 cells. **b**, The RNA levels of circp53 and linear p53 were determined by RT–qPCR with and without RNase R treatment in RKO, HCT116, HepG2, SGC-7901, A549 and MCF-7 cells. **c**, The levels of circp53 were significantly lower in patients with CRC compared with NP, as evaluated by RNAscope analysis. Representative staining images are shown, with positive reactions indicated by red arrows. **d**, Statistical analysis of RNAscope analysis *(P* < 0.01) .**e**, RNA levels of circp53 were determined by RT–PCR. **f**, WB analysis was performed to examine overexpression of circp53–209aa in RKO, HCT116, HepG2, SGC-7901, A549 and MCF-7 cells using the HA-tag antibody. **g**, The specific peptides from circp53–209aa were identified by MS analysis. **h** The MTT assay demonstrated decreased cell proliferation rates of circp53-OE RKO, HCT116 cells compared with Ctrl cells. **i**, The MTT assay demonstrated decreased cell proliferation rates of circp53-OE HepG2, SGC-7901 cells compared with Ctrl cells. **j**, The MTT assay demonstrated decreased cell proliferation rates of circp53-OE A549 and MCF-7 cells compared with Ctrl cells. **k** The EdU incorporation assay demonstrated the numbers of proliferating cells were significantly decreased in circp53–209aa-OE cells compared with Ctrl cells. **l**, Statistical analysis of EdU incorporation assay. **m**, The effects of circp53 overexpression on cell apoptosis in RKO, HCT116, HepG2, SGC-7901, A549 and MCF-7 cells. **n**, Statistical analysis of cell apoptosis. **o**, The TUNEL assay showed the numbers of apoptotic cells were significantly increased in circp53–209aa-OE cells compared with Ctrl cells. **p**, Statistical analysis of TUNEL assay. **q**, Flow cytometry showed an increased level of circp53–209aa, resulting in a decrease in the ATP level in cancer cells. **r**, Confocal microscopic analysis revealed a rapid and almost complete loss of Calcein fluorescence signal in A549, HepG2, SGC-7901 and MCF-7 circp53–209aa-OE cells. **s**, Statistical analysis of confocal microscopic analysis in A549, HepG2, SGC-7901 and MCF-7 circp53–209aa-OE cells. **t**, Confocal microscopic analysis revealed a rapid and almost complete loss of Calcein fluorescence signal in RKO and HCT116 circp53–209aa-OE cells. **u**, Statistical analysis of confocal microscopic analysis in RKO and HCT116 circp53–209aa-OE cells. **v,** The effects of circp53 overexpression on the mitochondrial apoptotic pathway associated proteins Bad, Bak, Bcl-xL, Bax and Bcl-2 in RKO and HCT116 cells. **w**, The effects of circp53 overexpression on the mitochondrial apoptotic pathway associated proteins Bcl-xL and Bcl-2 in HepG2 and SGC-7901 cells. **x**, Statistical analysis of expression of mitochondrial apoptotic pathway associated proteins Bad, Bak, Bcl-xL, Bax and Bcl-2 in RKO and HCT116 cells. **y**, Statistical analysis of expression of mitochondrial apoptotic pathway associated proteins Bad, Bak, Bcl-xL, Bax and Bcl-2 in HepG2 and SGC-7901 cells. The data are presented as mean ± s.d. (*n* = 6 clinical samples for each group, *n* = 3 cultures for each group, *P < 0.05, **P < 0.01 and ***P < 0.001).

The four most prevalent cancers worldwide are lung, gastric, hepatic and breast cancers. To explore the broader relevance of our findings across multiple cancer types, we first analyzed the expression levels of circp53 in six human cancer cell lines: two CRC cell lines (RKO and HCT116), a lung adenocarcinoma cell line (A549), a gastric cancer cell line (SGC-7901), a hepatoma cell line (HepG2) and a breast cancer cell line (MCF-7). As illustrated in Fig. 5a, b, circp53 was expressed in all six cancer cell lines and exhibited resistance to RNase R digestion. The circp53 abundance was significantly higher in NP tissues compared with tissues from patients with CRC (Fig. 5c,d). WB analysis confirmed the presence of circp53–209aa at the expected molecular weight in RKO and HCT116 cells (Supplementary Fig. 6a). To further investigate its functional role, we transduced CRC cells with circp53-OE plasmids and validated the overexpression using RT–qPCR, which demonstrated a marked increase in circp53 levels in RKO and HCT116 circp53-OE cells relative to Ctrl cells (Fig. 5e). Similarly, circp53 was overexpressed in HepG2, SGC-7901, A549 and MCF-7 cells. Subsequent WB and MS analyses further identified specific peptide fragments derived from circp53–209aa (Fig. 5f, g). Immunofluorescence staining revealed that circp53–209aa was predominantly localized in the cytoplasm, as detected by the HA antibody (Supplementary Fig. 6b). The MTT assay showed that elevated circp53–209aa expression significantly suppressed cellular proliferation across multiple cancer cell lines (Fig. 5h–j). The EdU incorporation assay also demonstrated that circp53–209aa-OE cells had a lower number of proliferating cells compared with Ctrl cells (Fig. 5k, l). Furthermore, an increased cell apoptosis rate was also observed in circp53–209aa-OE cells compared with Ctrl cells (Fig. 5m, n). This was further confirmed by the TUNEL assay, which revealed a higher level of apoptosis in circp53–209aa-OE cells compared with Ctrl cells (Fig. 5o, p). In addition, we observed that an increased level of circp53–209aa resulted in a decrease in the ATP level in cancer cells (Fig. 5q).

We performed Co-IP and immunofluorescence colocalization assays to elucidate the mechanism underlying circp53-induced apoptosis, demonstrating a specific interaction between circp53–209aa and CypD (Supplementary Fig. 6c,d). Subsequently, we employed the fluorescent dye calcein to assess the effect of circp53–209aa on mPTP function. Confocal microscopy and flow cytometry analyses revealed a rapid and nearly complete dissipation of the fluorescence signal in multiple circp53–209aa-OE cell lines, including HepG2, SGC-7901, RKO, HCT116, A549 and MCF-7 (Fig. 5r–u). Furthermore, the expression levels of Bcl-2 and Bcl-xL were markedly decreased, while the expression levels of Bak, Bad and Bax were significantly increased in circp53–209aa-OE cells compared with Ctrl cells (Fig. 5v–y). These findings suggest that circp53 levels are reduced in patients with cancer, and increasing circp53 levels activate the mitochondrial apoptosis pathway in various types of cancer.

### Construction of the E7-circp53-EV and Her2-circp53-EV delivery systems specifically targets BMSCs and CRC cells, respectively

**Fig. 6:**
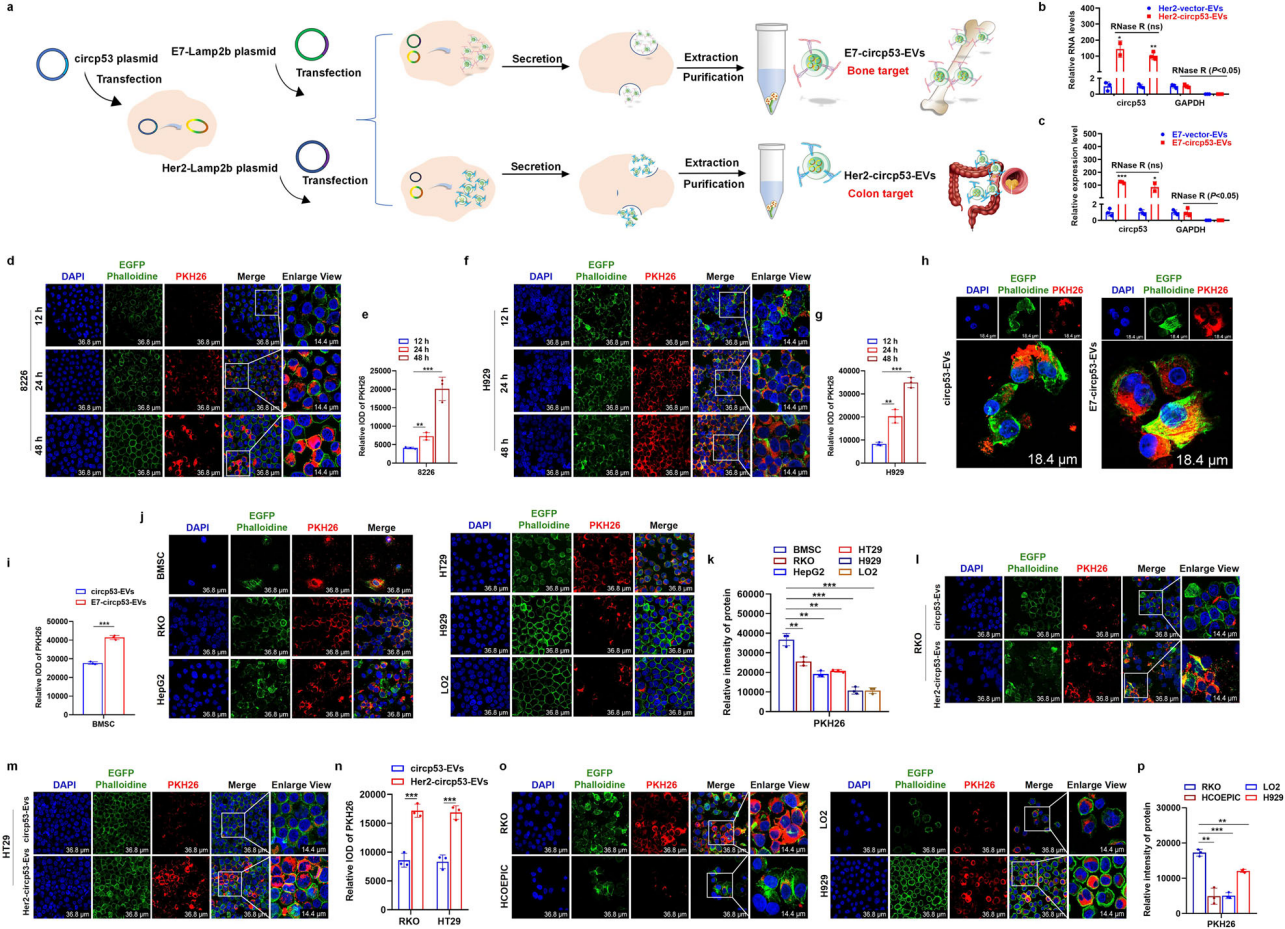
Construction of the E7-circp53-EV and Her2-circp53-EV delivery systems specifically targeting BMSCs and CRC cells, respectively. **a**, A schematic representation of the production and collection process of E7-circp53-EVs and Her2-circp53-EVs for targeted circp53 delivery. **b**, Total RNA extracted from Her2-circp53-EVs was incubated with or without RNase R, followed by RT–qPCR analysis. **c**, Total RNA extracted from E7-circp53-EVs was incubated with or without RNase R, followed by RT–qPCR analysis. RNase R treatment decreased the linear RNA level of GAPDH, but did not affect circp53. **d**, Laser-scanning confocal microscopy was used to record the uptake of PKH26-labeled EVs (red) by RPMI 8226 cells (green). **e**, Statistical analysis of Laser-scanning confocal microscopy of RPMI 8226 cells. **f**, Laser-scanning confocal microscopy was used to record the uptake of PKH26-labeled EVs (red) by H929 cells (green). **g**, Statistical analysis of Laser-scanning confocal microscopy of H929 cells. **h**, Laser-scanning confocal microscopy was used to record the greater uptake of E7-circp53-EVs compared with circp53-EVs in BMSCs, demonstrating a high specific affinity of the E7 peptide sequence to BMSCs. **i**, Statistical analysis of Laser-scanning confocal microscopy of BMSCs. **j**, Laser-scanning confocal microscopy was used to record the greater uptake of E7-circp53-EVs in BMSC cells compared with RKO, HepG2, HT29, H929 and LO2 cells. **k**, Statistical analysis of Laser-scanning confocal microscopy of RKO, HepG2, HT29, H929 and LO2 cells. **l**, Laser-scanning confocal microscopy was used to record the greater uptake of Her2-circp53-EVs compared with circp53-EVs in RKO, confirming the ability of Her2-circp53-EVs to target CRC cells. **m**, Laser-scanning confocal microscopy was used to record the greater uptake of Her2-circp53-EVs compared with circp53-EVs in HT29 cells. **n**, Statistical analysis of Laser-scanning confocal microscopy of RKO and HT29 cells. **o**, Laser-scanning confocal microscopy was used to record the greater uptake of Her2-circp53-EVs in RKO cells compared with H929, LO2 and HCOEPIC cells. **p**, Statistical analysis of Laser-scanning confocal microscopy of EVs marked by PKH26. The data are presented as mean ± s.d. (*n* = 3 cultures for each group, *P < 0.05, **P < 0.01 and ***P < 0.001).

To validate the potential of EV-based nucleic acid drugs in treating cancer, two drug delivery systems were developed: E7-circp53-EVs for targeting human bone marrow-derived mesenchymal stem cells (hBMMSCs)^32^ and Her2-circp53-EVs for targeting CRC cells^33^ (Fig. 6a). The E7 peptide (EPLQLKM) was previously designated as an affinity peptide for hBMMSCs. The Her2-Lamp2b fusion protein was designed to facilitate targeted cellular uptake through epidermal growth factor receptor (EGFR)-mediated endocytosis in CRC cells. HEK293 cells were transduced with plasmids encoding E7-Lamp2b/Her2-Lamp2b and circp53, and EVs were subsequently purified (Fig. 6a). The EVs isolated from the cell culture supernatant were characterized using NTA, TEM and WB analyses. Both E7-circp53-EVs and Her2-circp53-EVs exhibited uniform physical properties, with NTA and TEM revealing peak diameter distributions at 120.2 nm and 115.5 nm, respectively (Supplementary Fig. 7a). Zeta potential measurements demonstrated highly negative surface charges of −49 mV and −48.8 mV for E7-circp53-EVs and Her2-circp53-EVs, respectively (Supplementary Fig. 7b). This strong negative charge confers enhanced colloidal stability by preventing EV aggregation and improving storage longevity. WB analysis confirmed the presence of canonical EV markers Alix and CD9, validating successful EV isolation (Supplementary Fig. 7c). Notably, quantitative analysis revealed significantly higher circp53 levels in E7-Lamp2b- and Her2-Lamp2b-expressing cells compared with the Ctrl group, indicating efficient circp53 incorporation into HEK293-derived EVs (Fig. 6b, c). Furthermore, RNase R resistance assays confirmed the structural stability of the overexpressed circp53 within the EVs (Fig. 6b, c).

The purified EVs were labeled with the well-recognized fluorescent membrane dye PKH26 (red fluorescence) to confirm their ability to be internalized by the cells. After incubation with PKH26-labeled E7-circp53-EVs for 12, 24 and 48 h, a punctuated pattern of green fluorescence was observed in RPMI 8226 and H929 cells, with the signal increasing over time (Fig. 6d–g). Furthermore, after 24 h of incubation with PKH26-labeled EVs, bone mesenchymal stem cells (BMSCs) showed a higher uptake of E7-circp53-EVs compared with circp53-EVs, indicating a high specific affinity of the E7 peptide sequence for BMSCs (Fig. 6h, i). To determine the specific cellular uptake, PKH26-labeled E7-circp53-EVs were then further incubated with BMSCs, RKO, HepG2, HT29, H929 and LO2 cells. Notably, BMSCs showed a significantly higher fluorescence intensity compared with other cells, further confirming the high affinity of the E7 peptide for BMSCs (Fig. 6j, k). Additionally, when PKH26-labeled circp53-EVs/Her2-circp53-EVs were incubated with RKO and HT29 cells, the cellular uptake of Her2-circp53-EVs was significantly higher than circp53-EVs, as observed by confocal microscopy (Fig. 6l–n). To examine whether Her2-circp53-EVs presented selectivity to CRC cells, PKH26-labeled Her2-circp53-EVs were then incubated with RKO, HCOEPIC, LO2 and H929 cells to determine the cellular uptake. A significantly higher fluorescence intensity was observed in RKO cells compared with other cells (Fig. 6o, p), further demonstrating a high targeting capability of Her2-circp53-EVs for CRC cells. These experiments suggest that the E7-circp53-EVs and Her2-circp53-EVs delivery systems can be developed to selectively target BMSC and CRC cells, respectively.

### E7-circp53-EVs and Her2-circp53-EVs selectively inhibit tumor growth in PDX and NOD/SCID-TIBIA mouse models

**Fig. 7:**
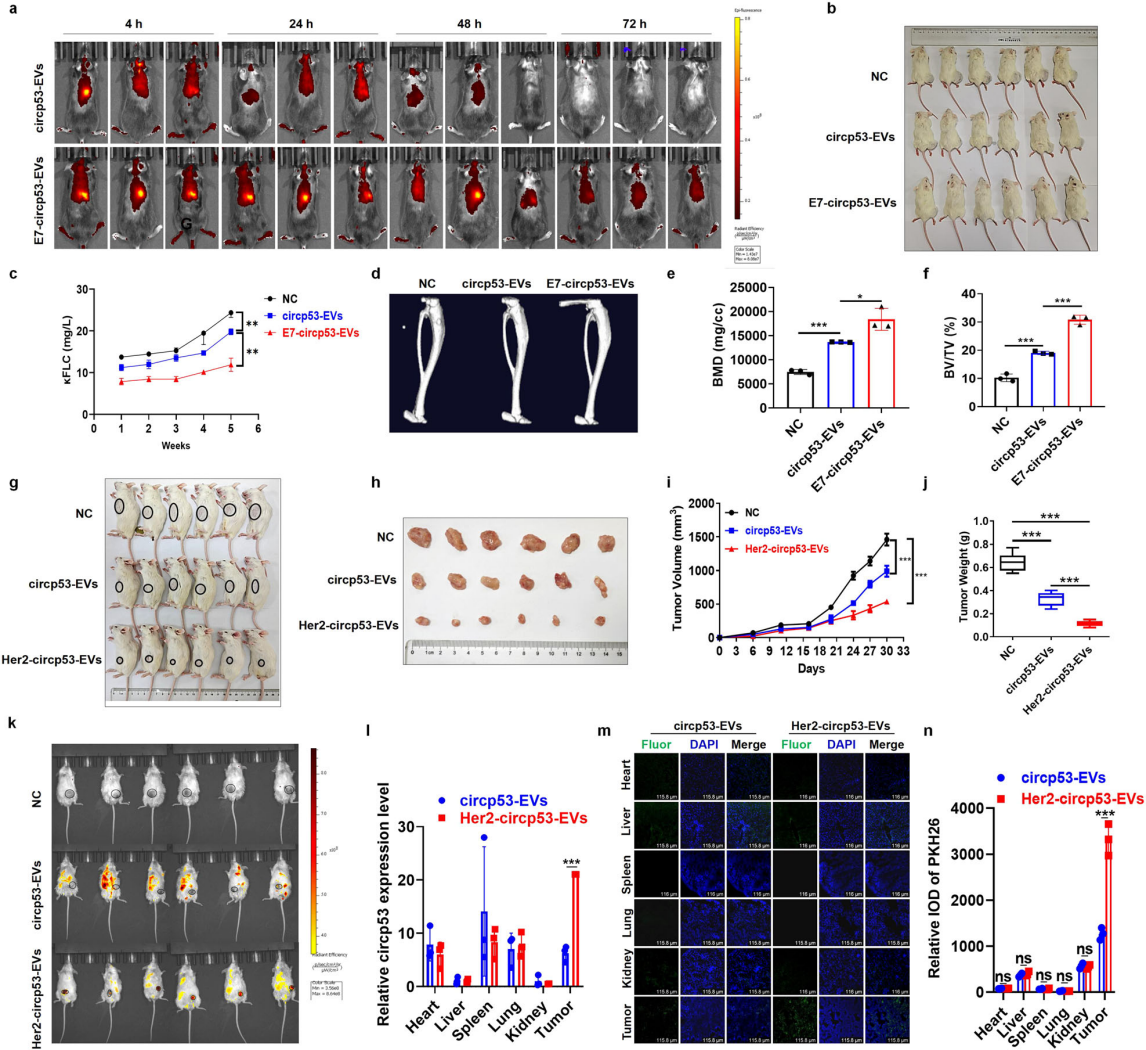
E7-circp53-EVs and Her2-circp53-EVs selectively inhibit tumor growth in PDX and NOD/SCID-TIBIA mouse models. **a**, An IVIS spectrum was recorded to show the high fluorescence intensity in the spine and prolonged retention time in the E7-circp53-EVs group, further confirming its ability to target bone in vivo. **b**, Images of NOD/SCID-TIBIA mice in NC, circp53-EVs and E7-circp53-EVs groups. **c**, Human κFLC levels in mouse serum were measured by an ELISA. **d**, Representative micro-computed tomography images of bones in the NC, circp53-EVs and E7-circp53-EVs groups. **e**, The BMD of NOD/SCID-TIBIA mice in the NC, circp53-EVs, and E7-circp53-EVs groups. **f**, The BV/TV of NOD/SCID-TIBIA mice in the NC, circp53-EVs and E7-circp53-EVs groups. **g**, **h**, Images of PDX model mice on day 27 (**g**) and tumors taken from mice in each group (**h**) (*P* < 0.001). **i**, Tumor growth curves of the PDX model for the NC, circp53-EVs and Her2-circp53-EVs groups. **j**, Tumor weights of the PDX model in the NC, circp53-EVs and E7-circp53-EVs groups. **k**, IVIS was employed to observe the fluorescence in the NC, circp53-EVs and E7-circp53-EVs groups. **l**, RT–qPCR analysis revealed that Her2-circp53-EVs accumulated to a greater extent in PDX tumors compared with other organs such as the heart, liver, spleen, lung and kidney. **m**, Confocal imaging of EVs biodistribution in murine organs. **n**, Confocal microscopy of frozen sections showed that Her2-circp53-EVs accumulated to a greater extent in PDX tumors compared with other organs such as the heart, liver, spleen, lung and kidney by quantitative statistics. The data are presented as mean ± s.d. (*n* = 6 mice for each group, ^*^P < 0.05, ^**^P < 0.01, ^***^P < 0.001 and ns, no statistical significance).

Dil-labeled circp53-EVs and E7-circp53-EVs were respectively administered to mice via tail vein injection, and their targeting capabilities were subsequently evaluated using IVIS (Spectrum; PerkinElmer, Inc.). Interestingly, comparatively higher fluorescence intensities in the spine and longer retention times were observed in the E7-circp53-EV group compared with the circp53-EV group (Fig. 7a). To further validate the targeting ability of E7-circp53-EVs, RPMI 8226 WT cells (1 × 10^7^/10 μl) were injected into the bone marrow cavity of the tibias in 6–8-week-old NOD/SCID mice, which were randomly divided into three groups (Fig. 7b). The tumor burden was measured by detecting the blood kappa free light chain (κFLC) levels using an enzyme-linked immunosorbent assay (ELISA) κFLC detection kit (Yifei Xue Bio Tech Ltd.). Osteolysis, a biomarker of tumor burden in MM, can be reflected by the serum level of κFLC^34^. The κFLC level was significantly lower in E7-circp53-EVs group compared with nontreated and circp53-EVs groups (*P* < 0.01) (Fig. 7c). Micro-computed tomography imaging of the bones subsequently showed that the level of bone damage was significantly reduced in E7-circp53-EVs group compared with nontreated and circp53-EVs groups at the experimental endpoint (Fig. 7d). Additionally, bone mineral density (BMD) and the bone volume fraction (BV/TV) were also measured and found to be significantly higher in E7-circp53-EVs group compared with nontreated and circp53-EVs groups (Fig. 7e, f). These results confirm the bone-targeting ability of E7-circp53-EVs in vivo.

**Fig. 8:**
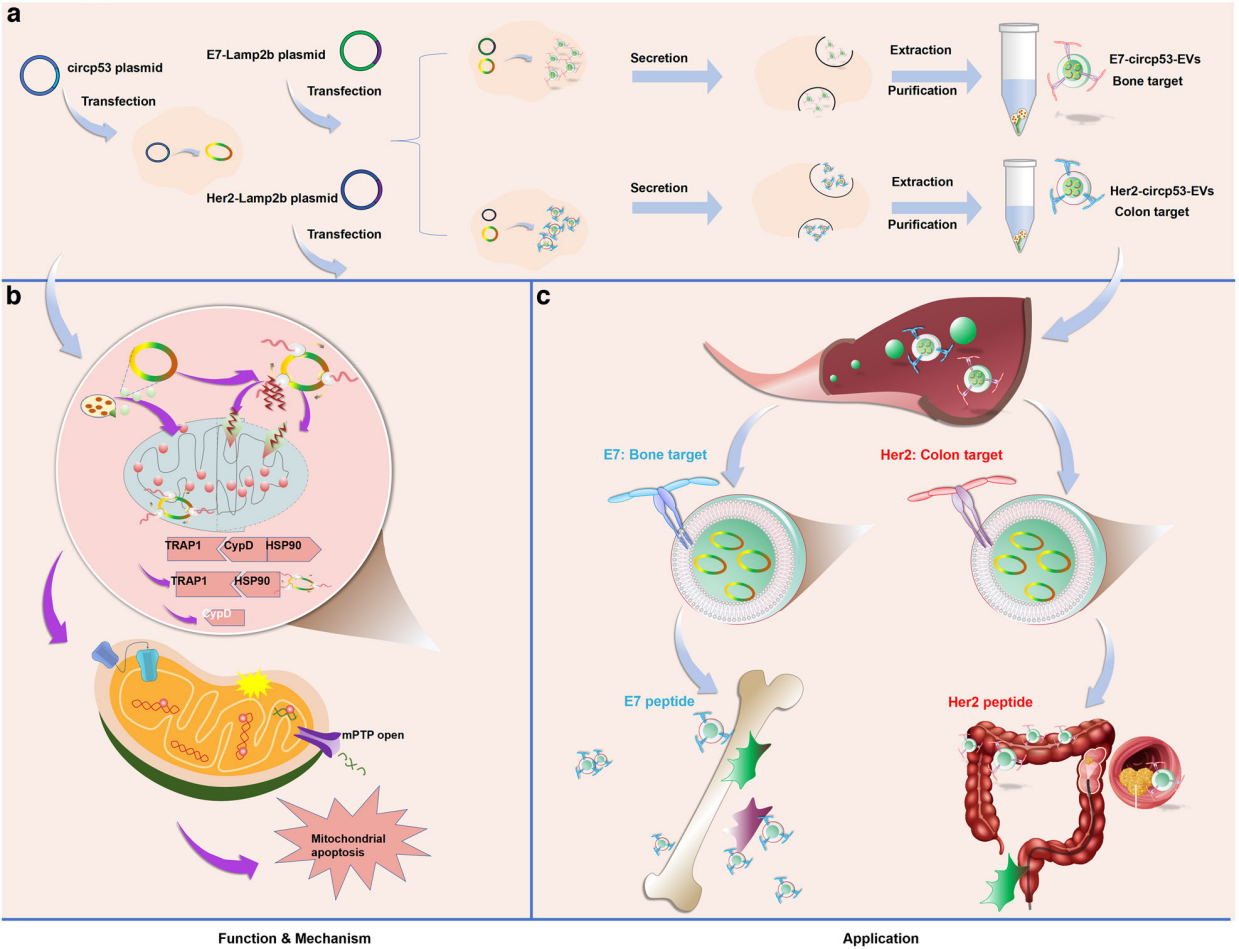
A schematic diagram illustrates how the targeted delivery of circp53 via EVs suppresses cancer progression by opening the mPTP. **a**, The process of packaging tumor-targeted EVs that overexpress circp53. **b**, The mechanism of tumor cell cytotoxicity mediated by EVs that overexpress circp53. **c**, The targeted delivery of circp53-enriched EVs to CRC and MM.

To extend our exploration of the functions of Her2-circp53-EVs in vivo, we utilized a CRC PDX model. Circp53-EVs and Her2-circp53-EVs were administered intravenously via tail injection in PDX model mice. The tumors in the Her2-circp53-EVs group were significantly smaller than those in the circp53-EVs group (Fig. 7g, h). The average volume and weight of tumors in the Her2-circp53-EVs group were substantially lower than the tumors in the circp53-EVs group (Fig. 7I, j) (*P* < 0.001). Then, we used IVIS to track the biodistribution of Her2-circp53-EVs in vivo. A higher fluorescence intensity was observed in the tumors of Her2-circp53-EVs mice compared with those in Ctrl and circp53-EVs mice (Fig. 7k). Consistently, RT–qPCR analysis and confocal microscopic observation of the frozen sections showed that Her2-circp53-EVs accumulated to a greater extent in PDX tumors than they did in other organs (that is, the heart, liver, spleen, lung and kidney) compared with circp53-EVs (Fig. 7l–n). These findings suggest that engineered E7-circp53-EVs and Her2-circp53-EVs may serve as novel targeted therapies with high potential for treating refractory cancer in the future (Fig. 8).

## Discussion

Mutations in the *TP53* gene are frequent events in cancer, and therapeutic strategies to restore p53 function have been explored for decades^5^. However, only a few drug development programs have made it to late-stage clinical trials and currently there are no p53-based treatments approved in the USA or Europe. As a nuclear transcription factor, *TP53* does not possess the typical characteristics of a drug target and for a long time it was believed to be impossible to target^35^. In 1999, Pfizer developed the first compound with the ability to reactivate mutated *TP53* (CP31398), which showed promising results in restoring the transcriptional activity of *TP53* and reducing tumor growth in animal studies^36^. However, the side effects of CP31398, such as nonspecific toxicity caused by binding to DNA, have prevented it from being further developed for clinical use. As a result, many researchers have turned their attention to inhibitors of mouse double minute 2 homolog (MDM2). The first inhibitors were nutlins, which have been shown to activate *TP53* in cancer cells with normal p53 protein. However, these inhibitors have also been found to have substantial side effects, including gastrointestinal issues, suppression of blood cell production and low blood cell counts^37^. On a different note, gene therapy has recently gained renewed interest. Gendicine, a recombinant human *TP53* adenovirus developed by Shenzhen SiBiono GeneTech Co. Ltd., was approved by the China Food and Drug Administration in 2003 for treating head and neck squamous cell carcinoma. Since then, gendicine has been administered to thousands of patients in China and has been reported to achieve notablely higher response rates when combined with chemotherapy or radiotherapy compared with standard treatments^38^.

Although major progress has been made in p53-based cancer therapies, there are still many challenges to overcome in the search for highly effective and selective drugs that can be used in clinical settings. One major concern regarding p53-based therapy is the emergence of resistance, which is a common issue with all anticancer treatments. Recent studies have highlighted the therapeutic potential of circRNAs in prolonged expression and translation mode, making them a promising therapeutic option. In the present study, we focused on the circular form of the *TP53* gene, and discovered that its coding product, circp53–209aa, can competitively interact with CypD from the CypD/TRAP1/HSP90 complex, leading to the activation of its isomerase activity and the induction of mPTP opening in both *TP53* mutant and nonmutated tumor cells. Of note, the WT p53 protein also has additional anticancer functions at the mitochondrial level, such as driving transcription-independent apoptosis^39^. Apart from p53, a few extra-mitochondrial proteins have been identified to be associated with CypD or involved in pathological mPTP opening. Our study focused on the structural and functional details of the mPTP, specifically the regulation of this process^40^. The regulation of the mPTP has been recognized as a potential therapeutic target for various diseases, including ischemia–reperfusion injury, Alzheimer’s disease and dementia and cancer^41^. The potential of circRNAs as therapeutic agents is now better understood, and there is growing evidence that they play essential roles in regulating gene flow in the nucleus and cytoplasm, potentially leading to gene reprogramming. For example, Li et al. reported that circACC1 could be an economic means to elicit AMPK activation and proposed that cancer cells exploit circACC1 during metabolic reprogramming^42^. Recently, Chen et al. unveiled that upregulated circRAD18 promotes tumor progression by reprogramming glucose metabolism in papillary thyroid cancer^43^. Our work demonstrates that circp53–209aa may offer a novel approach for p53 reprogramming, which can be particularly effective for high-risk, relapsed and p53 missense-mutation cancers.

The Keats Laboratory at TGen identified *TP53* mutations in hematological malignancy cell lines XG1 (c.377 A > T, p.Y126N) and RPMI 8226 (c.853 G > A, p.E285K). While these mutations are documented, their impact on circp53 formation, a circRNA derived from *TP53* back splicing, remains unexplored. Circp53 biogenesis depends on three factors: (1) intronic complementary sequences enabling exon circularization, (2) RNA-binding proteins regulating splicing efficiency and (3) linear *TP53* mRNA substrate availability^44^. *TP53* mutations may disrupt circp53 production through multiple mechanisms. Mutations in intronic complementary sequence regions could impair the complementary base pairing required for back splicing, as seen with the R273C mutation disrupting exon 6/7 splicing signals^45,46^. Structural alterations in *TP53* mRNA might destabilize RNA-binding protein interactions (for example, Quaking protein) critical for circularization^47,48^. Paradoxically, nonsense mutations may stabilize linear *TP53* mRNA by evading nonsense-mediated decay, creating substrate competition that inhibits circRNA production^49^. Aberrant splicing patterns from *TP53* mutations (exon skipping and cryptic splice sites) could alter the exon composition and functional domains of circp53^50^. Mutation-induced m6A methylation changes might further disrupt circp53 regulation^51^. Building upon mechanistic insights from referenced studies, we postulate that hematopoietic cell lines XG1 and RPMI 8226 exhibit an inherent absence of detectable circp53 expression, a phenomenon potentially attributable to their characteristic *TP53* mutational profiles that may comprehensively compromise the molecular framework essential for competent circRNA biogenesis.

Encouraged by the success of using EVs to deliver siRNAs and miRNAs, we preloaded circp53 into EVs. To avoid systemic side effects and to achieve active targeted delivery, we modified EVs with Lamp2b-E7 to target hBMMSCs and alternatively with Lamp2b-Her2 to target CRC cells, to release circp53 at the tumor site with spatial and temporal selectivity using the affinity peptide. In the future, it will be of great interest to utilize other surface-modification ligands to target various high-risk cancers, such as Lamp2b-VEGF for liver cancer, Lamp2b-EGFR for lung cancer, Lamp2b-RVG for neuron cancer and Lamp2b-MG7 for stomach cancer. However, several shortcomings of EVs need to be addressed and optimized. First, the translation efficiency of circRNAs needs to be improved^52^. We plan to explore various approaches to accomplish this goal, including optimizing key mediators of cap-independent translation such as internal ribosome entry site, epigenetic modification, m6A modification, acetylation and optimization of the 5′- and 3′-untranslated regions. These strategies should help improve circRNA translation and enhance the treatment of high-risk cancer. Furthermore, the electroporation approach is of great importance for improving the transfection efficiency. Second, the delivery methods and systems of circRNAs also need to be optimized. CircRNAs could be encapsulated, for example, within microspheres, liposomes, vesicles, albumin and other formulations such as freeze-dried powder to prevent leakage of the therapeutic cargo. One intriguing possibility is to attempt to induce controllable and predictable self-assembly and delivery of circRNAs in a heterogeneous, dynamic in vivo environment, allowing for the precise control of gene expression in a targeted manner. Third, the particle size of EVs should be reduced to achieve better tumor-targeting ability. The accumulation of EVs in the liver in our study may be due to immunorecognition of EVs caused by trans-endothelial transport, vascular fenestrations and uptake by Kupffer cells. The development of PEGylated nanomedicines is of great importance, such as Doxil (Sequus Pharmaceuticals Inc.), a PEGylated liposomal product of doxorubicin in clinical use that has been developed with a diameter <100 nm and shown to have greater efficacy. In our study, E7-Lamp2b-EVs were physically homogeneous, with a peak size of 120.2 nm in diameter, as determined by NTA. Chemical modifications, such as PEG modification on the surface of EVs, can prevent vesicle aggregation and reduce the particle size, leading to decreased liver uptake and improved tumor-targeting ability. Fourth, although EVs with targeted chemical modifications on their surface can penetrate the tumor microenvironment and accumulate at the tumor site, the RNAs released from EVs may still be easily degraded in endo/lysosomes after cellular uptake by endocytosis. Therefore, it is crucial to develop endo/lysosomal methods to effectively release RNAs into the cytosol for translation into functional proteins. For example, Xu et al. developed pH-activated nanoparticles for the augmented cytosolic delivery of POLR2A siRNA (siPol2), thereby improving the endo/lysosomal escape of siRNA. Overall, while there is promising preclinical evidence for the ability of systemically injected EVs to reach tumor sites, further research is needed to fully understand the biogenesis, cargo sorting, subpopulations, internalization and trafficking pathways of EVs in recipient cells, which is necessary to optimize EVs as drug delivery systems^53^.

In conclusion, our study demonstrates that circp53 overexpression suppresses proliferation and induces apoptosis across multiple tumor cell lines. Furthermore, we establish Her2-circp53-EVs and E7-circp53-EVs as a novel delivery platform capable of spatiotemporal-specific tumor targeting. This system significantly enhances apoptosis in both WT and mutated p53 cancer cells through a mechanism involving the circp53-encoded functional peptide circp53–209aa. Mechanistically, circp53–209aa competitively disrupts the CypD/TRAP1/HSP90 complex, activating the isomerase activity of CypD and triggering mPTP opening. This therapeutic strategy holds potential for extension to other malignancies, including colorectal, breast and non-small-cell lung cancers. Collectively, our findings validate engineered EVs as a multifunctional nanotherapeutic platform for precision oncology. The translational-circRNA-EVs system developed in this work offers a promising therapeutic strategy for cancers driven by high-risk genetic mutations, especially those resistant to conventional therapies. These advancements underscore the broad clinical potential of EV-based platforms in addressing unmet needs in the treatment of aggressive and treatment-resistant cancers.

### The Paper Explained

#### Problem

Despite significant advancements in novel drugs and treatment methods, including cellular therapy, the majority of cancers remain incurable. Certain cancers with high-risk genetic mutations, such as p53, often show little response to traditional treatments. However, the success of mRNA vaccines has sparked interest in exploring the potential of circular RNA (circRNA) drugs.

## Results

Using multiple myeloma (MM) as a working model, we first identified a circRNA form of the *TP53* gene, hsa_circp53_0041947. This form was found to be significantly decreased in patients with MM and was also associated with potent anti-MM effects. The functional peptide encoded by hsa_circp53_0041947, named circp53–209aa, exhibited potent anti-MM effects in vitro and in vivo, regardless of p53 mutations. The underlying mechanism revealed that circp53–209aa can interact with cyclophilin D (CypD) from the CypD/TRAP1/HSP90 complex, activating the isomerase activity of CypD and inducing the opening of the mitochondrial permeability transition pore, ultimately leading to mitochondrial apoptosis. To further enhance the delivery of hsa_circp53_0041947, we developed a targeted extracellular vesicle (EV) delivery system. This system utilizes engineered E7-Lamp2b-EVs and Her2-Lamp2b-EVs delivery systems designed to target MM and colorectal cancer (CRC), respectively. The targeted delivery of hsa_circp53_0041947 achieved robust tumor inhibitory effects on both MM and CRC, suggesting its potential applicability for other cancers.

### Impact

Our findings not only demonstrate the effectiveness of circp53-EVs in treating various types of cancer, but also showcase the potential of this engineered circRNA-EVs delivery system as a promising circRNA-based therapeutic approach for high-risk and relapsed cancers.

## Supplementary information

Supplementary information

Supplementary Table 1

Supplementary Table 2

Supplementary Table 3

Supplementary Table 4

## Data Availability

All data associated with this study are present in the Article and its Supplementary Information. All raw data from our research are available upon reasonable request. The RNA-sequencing data were deposited in GEO (GSE248072). The URL address of the RNA-sequencing data is https://www.ncbi.nlm.nih.gov/geo/query/acc.cgi?acc=GSE248072. The following secure token has been created to allow the review of record GSE248072 while it remains in private status: wfmzggcmfhyldwp. The MS data can be found on the ProteomeXchange Consortium website: PXD047003. The URL address of the MS data is as follows: https://www.iprox.cn/page/SSV024.html;url=1744777444887rWnD. Password: MNbi.
