# Supplementary material for: Extracellular vesicle-mediated delivery of circp53 suppresses the progression of multiple cancers by activating the CypD/TRAP/HSP90 pathway
Source: Exp Mol Med. 2025 Aug 1;57(8):1711–26. doi: 10.1038/s12276-025-01506-0 (PMC12411616; doi:10.1038/s12276-025-01506-0)
Supplement: Supplementary file 1 — Supplementary information [file 12276_2025_1506_MOESM1_ESM.pdf]

List of Supplementary Materials

Present a list of the Supplementary Materials in the following format.

Supplementary Fig. 1 to 7: Supplementary Figures demonstrate the targeted delivery of circp53 via extracellular vesicles, effectively inhibiting the progression of various types of cancer.

Other Supplementary Material for this manuscript includes the following items:

Supplementary Table 1 to 4: Supplementary tables demonstrate that the p53 signaling pathway is significantly upregulated upon circp53 overexpression in MM1.S, H929, RPMI 8226, and XG1 cells.

Supplementary Fig. 1

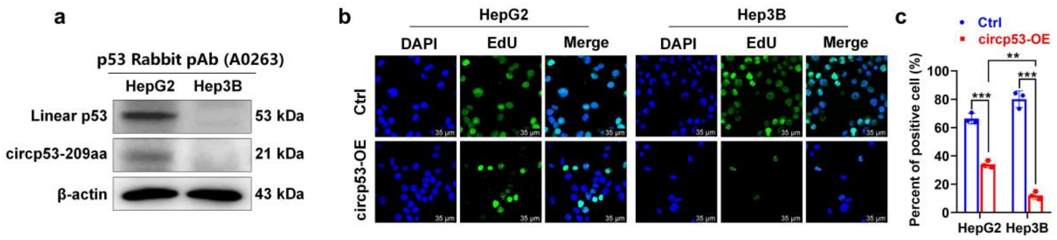

Supplementary Fig. 1 Validation of Hep3B cells as a suitable negative control. (**a**) WB analysis of linear p53 and circp53 in HepG2 (p53 WT) and Hep3B (p53-deleted) cells. (**b** and **c**) The EdU incorporation assay demonstrated that circp53 overexpression inhibited the proliferation of Hep3B cells more markedly than that of HepG2 cells. The data are presented as mean±SD. $^{*}P<0.05$; $^{**}P<0.01$; $^{***}P<0.001$; WT, wild-type; WB, western blotting; Ctrl, control; MUT, mutation; EdU, 5-Ethynyl-2'-deoxyuridin.

Supplementary Fig. 2

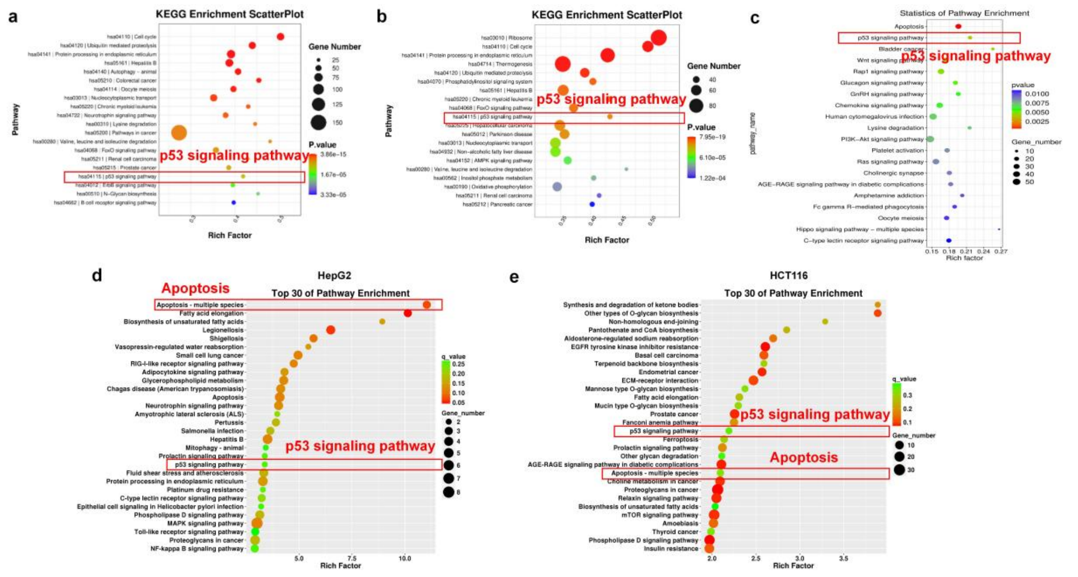

Supplementary Fig. 2 KEGG pathway enrichment analysis of RNA-seq data. A significant activation of the p53 signaling pathway exists in XG1 (**a**), MM1.S (**b**), H929 (**c**), HepG2 (**d**), and HCT116 (**e**) cell lines upon circp53 overexpression.

Supplementary Fig. 3

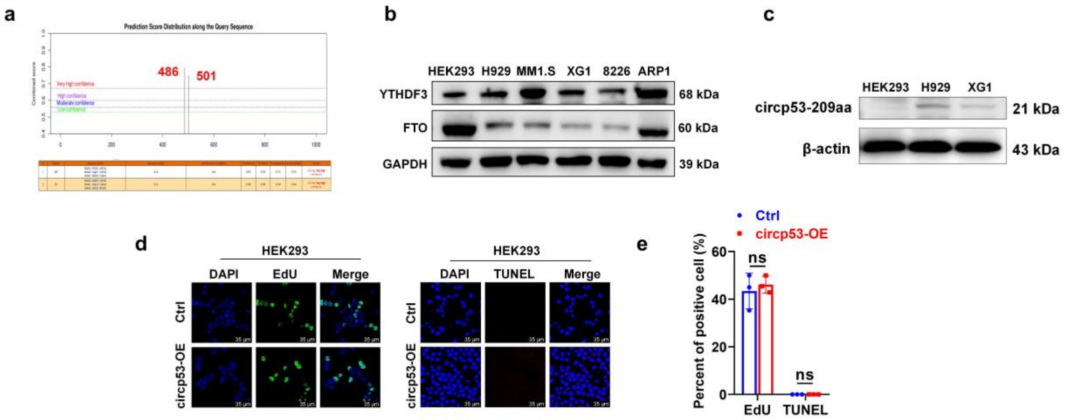

Supplementary Fig. 3 Construction and verification of plasmids with circp53 initiation codon mutation. (**a**) Prediction of m6A methylation site of circp53. (**b**) WB analysis was used to determine the methylation level of m6A in normal and tumor cells. (**c**) The expression of circp53-209aa was observed in different cells. (**d** and **e**) EdU incorporation and TUNEL assays were conducted to demonstrate the difference between Ctrl and circp53-OE HEK293 cells on cell proliferation and apoptosis. Scale bar, 35 μm. The data are presented as mean±SD. $^{*}P$<0.05; $^{**}P$<0.01; $^{***}P$<0.001; "ns" represents no statistical significance. Ctrl, control; MUT, mutation; EdU, 5-Ethynyl-2'-deoxyuridine; TUNEL, Terminal deoxynucleotidyl transferase-mediated dUTP-biotin nick end labeling.

Supplementary Fig. 4

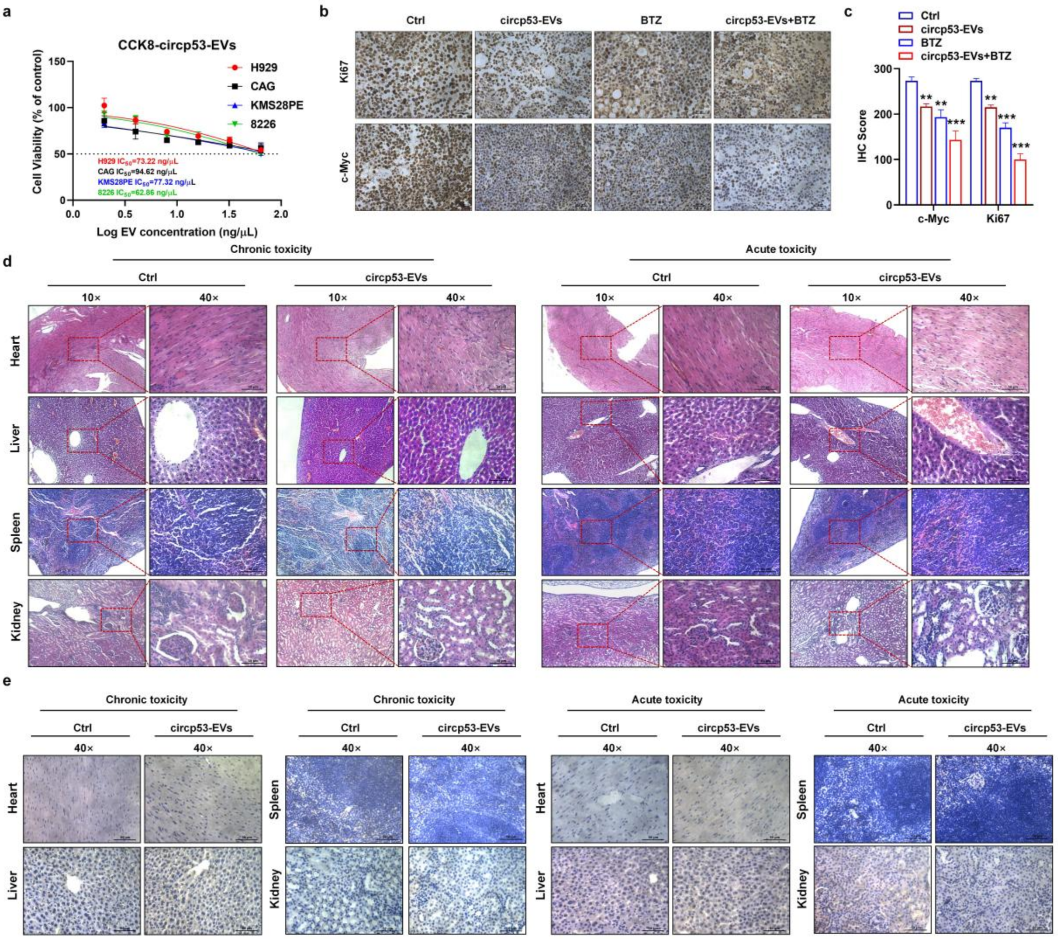

Supplementary Fig. 4 The impact of exosomes *in vivo*. (**a**) CCK8 analysis of IC_{50} of circp53-EVs on tumor cells. (**b** and **c**) The levels of Ki67 and c-Myc in the circp53-EVs, BTZ, and circp53+BTZ groups were significantly lower than those in the Ctrl group. Scale bar, 100 µm. (**d**) H&E staining results of various organs in mice. Scale bar, 50 µm. (**e**) IHC staining results of TNF-α, an inflammatory index in various organs of mice. scale bar, 50 µm. The data are presented as mean±SD. $^{*}P<0.05$; $^{**}P<0.01$; $^{***}P<0.001$. EV, extracellular vesicle; Ctrl, control; CCK-8, Cell Counting Kit-8; IHC, immunohistochemistry; H&E, hematoxylin-eosin.

Supplementary Fig. 5

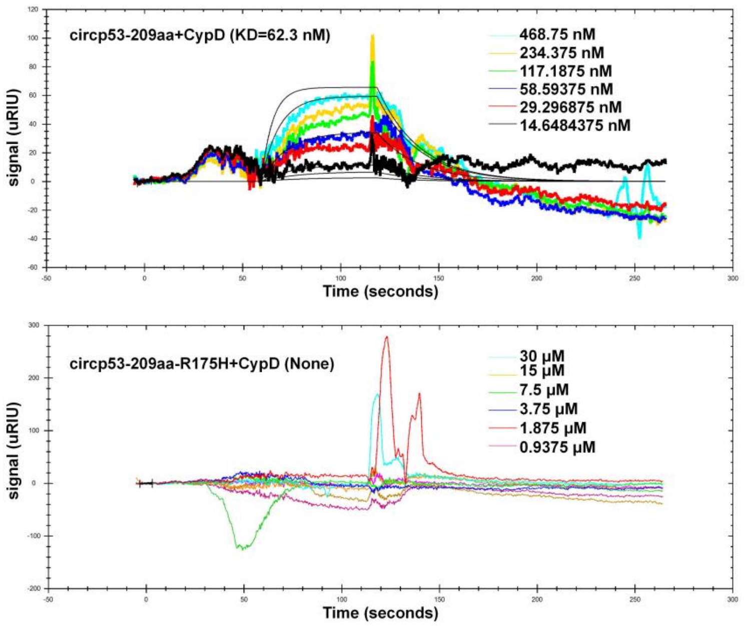

Supplementary Fig. 5 The binding affinity between varying concentrations of circp53-209aa or circp53-209aa-R175H and CypD protein was analyzed using the Reichert 4SPR molecular interaction instrument.

Supplementary Fig. 6

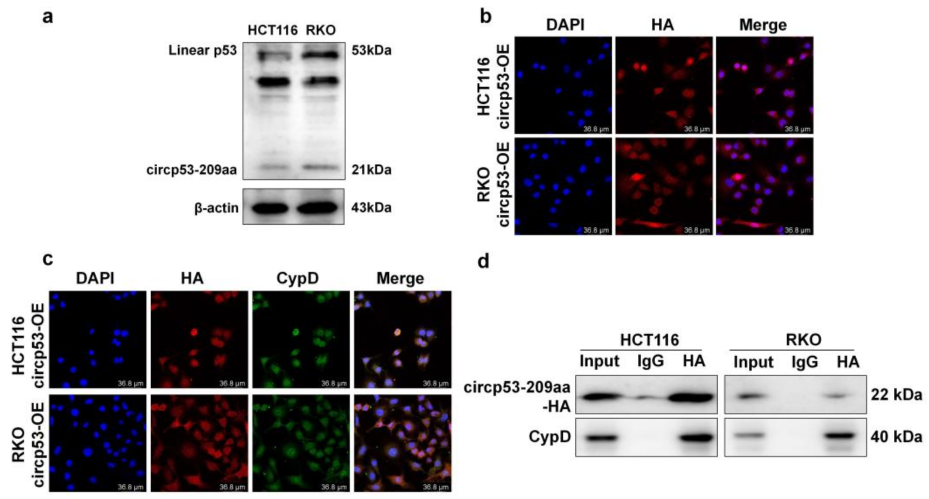

Supplementary Fig. 6 Circp53-209aa interacts with CypD in HCT116 and RKO cells. (**a**) WB analysis of endogenous circp53-209aa expression in HCT116 and RKO cells. (**b**) Live confocal microscope images were captured to demonstrate the cellular localization of circp53-209aa. Scale bar, 25 μm. (**c**) Representative confocal images for HA and CypD, indicating the interaction between circp53-209aa and CypD. (**d**) A Co-IP assay confirmed the direct interaction between circp53-209aa and CypD in HCT116 and RKO cells.

Supplementary Fig. 7

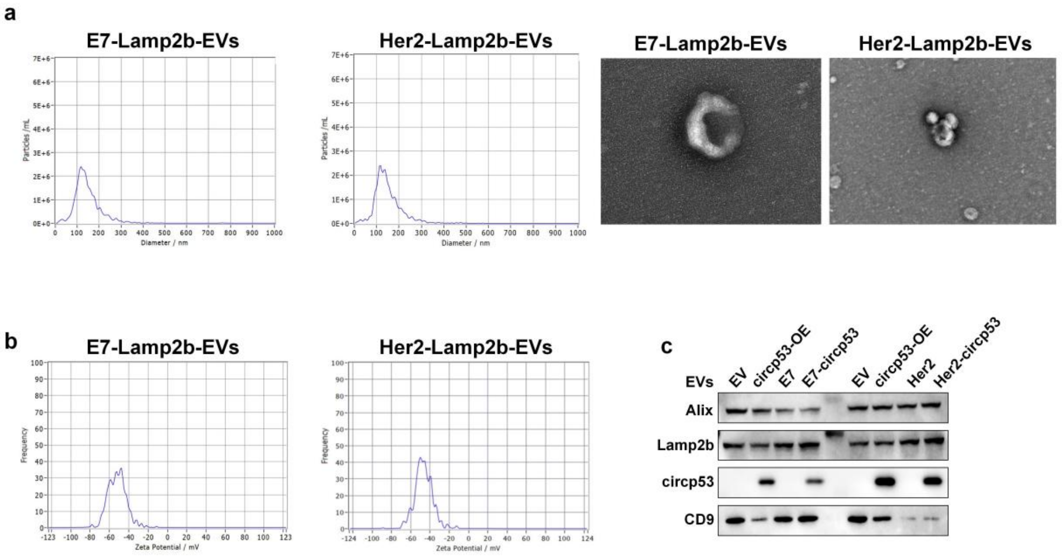

Supplementary Fig. 7 Physicochemical properties of E7-circp53-Evs and Her2-circp53-Evs. (**a**) Analyses of E7-circp53-EVs and Her2-circp53-EVs using nanoparticle tracking (NTA) and transmission electron microscopy (TEM). (**b**) Zeta potential analysis of E7-circp53-EVs and Her2-circp53-EVs. (**c**) WB analysis of classic EV markers, Alix and CD9.

Supplementary Tables

Supplementary Table 1 KEGG pathway enrichment analysis of RNA-seq data upon circp53 overexpression in MM1.S cells (Excel file).

Supplementary Table 2 KEGG pathway enrichment analysis of RNA-seq data upon circp53 overexpression in H929 cells (Excel file).

Supplementary Table 3 KEGG pathway enrichment analysis of RNA-seq data upon circp53 overexpression in RPMI 8226 cells (Excel file).

Supplementary Table 4 KEGG pathway enrichment analysis of RNA-seq data upon circp53 overexpression in XG1 cells (Excel file).
